# Embeddings from protein language models predict conservation and variant effects

**DOI:** 10.1007/s00439-021-02411-y

**Published:** 2021-12-30

**Authors:** Céline Marquet, Michael Heinzinger, Tobias Olenyi, Christian Dallago, Kyra Erckert, Michael Bernhofer, Dmitrii Nechaev, Burkhard Rost

**Affiliations:** 1 Department of Informatics, Bioinformatics and Computational Biology - i12, TUM-Technical University of Munich, Boltzmannstr. 3, Garching, 85748 Munich, Germany; 2 TUM Graduate School, Center of Doctoral Studies in Informatics and its Applications (CeDoSIA), Boltzmannstr. 11, 85748 Garching, Germany; 3 Institute for Advanced Study (TUM-IAS), Lichtenbergstr. 2a, Garching, 85748 Munich, Germany; 4 TUM School of Life Sciences Weihenstephan (TUM-WZW), Alte Akademie 8, Freising, Germany

## Abstract

The emergence of SARS-CoV-2 variants stressed the demand for tools allowing to interpret the effect of single amino acid variants (SAVs) on protein function. While Deep Mutational Scanning (DMS) sets continue to expand our understanding of the mutational landscape of single proteins, the results continue to challenge analyses. Protein Language Models (pLMs) use the latest deep learning (DL) algorithms to leverage growing databases of protein sequences. These methods learn to predict missing or masked amino acids from the context of entire sequence regions. Here, we used pLM representations (embeddings) to predict sequence conservation and SAV effects without multiple sequence alignments (MSAs). Embeddings alone predicted residue conservation almost as accurately from single sequences as ConSeq using MSAs (two-state Matthews Correlation Coefficient—MCC—for ProtT5 embeddings of 0.596 ± 0.006 vs. 0.608 ± 0.006 for ConSeq). Inputting the conservation prediction along with BLOSUM62 substitution scores and pLM mask reconstruction probabilities into a simplistic logistic regression (LR) ensemble for Variant Effect Score Prediction without Alignments (VESPA) predicted SAV effect magnitude without any optimization on DMS data. Comparing predictions for a standard set of 39 DMS experiments to other methods (incl. ESM-1v, DeepSequence, and GEMME) revealed our approach as competitive with the state-of-the-art (SOTA) methods using MSA input. No method outperformed all others, neither consistently nor statistically significantly, independently of the performance measure applied (Spearman and Pearson correlation). Finally, we investigated binary effect predictions on DMS experiments for four human proteins. Overall, embedding-based methods have become competitive with methods relying on MSAs for SAV effect prediction at a fraction of the costs in computing/energy. Our method predicted SAV effects for the entire human proteome (~ 20 k proteins) within 40 min on one Nvidia Quadro RTX 8000. All methods and data sets are freely available for local and online execution through bioembeddings.com, https://github.com/Rostlab/VESPA, and PredictProtein.

**Supplementary Information:**

The online version contains supplementary material available at 10.1007/s00439-021-02411-y.

## Introduction

**Many different resources capture SAV effects.** Mutations in the Spike (S) surface protein of SARS-CoV-2 have widened the attention to the complex issue of protein variant effects (Korber et al. 2020; Laha et al. 2020; Mercatelli and Giorgi 2020; O’Donoghue et al. 2020). The ability to distinguish between beneficial (= gain of function, GoF), deleterious (= loss of function, LoF) and neutral single amino acid variants (SAVs; also referred to as SAAV, missense mutations, or non-synonymous Single Nucleotide Variants: nsSNVs) continues to be a key challenge toward understanding how SAVs affect proteins (Adzhubei et al. 2010; Bromberg and Rost 2007, 2009; Ng and Henikoff 2003; Studer et al. 2013; Wang and Moult 2001). Recently, an unprecedented amount of in vitro data describing the quantitative effects of SAVs on protein function has been produced through Multiplexed Assays of Variant Effect (MAVEs), such as deep mutational scans (DMS) (Fowler and Fields 2014; Weile and Roth 2018). However, a comprehensive atlas of in vitro variant effects for the entire human proteome still remains out of reach (AVE Alliance Founding Members 2020). Yet, even for the existing experiments, intrinsic problems remain: (1) In vitro DMS data capture SAV effects upon molecular function much better than those upon biological processes, e.g., disease implications may be covered in databases such as the Online Mendelian Inheritance in Man (OMIM) (Amberger et al. 2019), but not in MaveDB (Esposito et al. 2019). (2) The vast majority of proteins have several structural domains (Liu and Rost 2003, 2004a, b); hence, most are likely to have several different molecular functions. However, each experimental assay tends to measure the impact upon only one of those functions. (3) In vivo protein function might be impacted in several ways not reproducible by in vitro assays.

**Evolutionary information from MSAs is most important to predict SAV effects.** Many in silico methods try to narrow the gap between known sequences and unknown SAV effects; these include (by earliest publication date): PolyPhen/PolyPhen2 (Adzhubei et al. 2010; Ramensky et al. 2002), SIFT (Ng and Henikoff 2003; Sim et al. 2012), I-Mutant (Capriotti et al. 2005), SNAP/SNAP2 (Bromberg and Rost 2007; Hecht et al. 2015), MutationTaster (Schwarz et al. 2010), Evolutionary Action (Katsonis and Lichtarge 2014), CADD (Kircher et al. 2014), PON-P2 (Niroula et al. 2015), INPS (Fariselli et al. 2015), Envision (Gray et al. 2018), DeepSequence (Riesselman et al. 2018), GEMME (Laine et al. 2019), ESM-1v (Meier et al. 2021), and methods predicting rheostat positions susceptible to gradual effects (Miller et al. 2017). Of these, only Envision and DeepSequence trained on DMS experiments. Most others trained on sparsely annotated data sets such as disease-causing SAVs from OMIM (Amberger et al. 2019), or from databases such as the protein mutant database (PMD) (Kawabata et al. 1999; Nishikawa et al. 1994). While only some methods use sophisticated algorithms from machine learning (ML; SVM, FNN) or even artificial intelligence (AI; CNN), almost all rely on evolutionary information derived from multiple sequence alignments (MSAs) to predict variant effects. The combination of evolutionary information (EI) and ML/AI has long been established as a backbone of computational biology (Rost 1996; Rost and Sander 1992, 1993), now even allowing AlphaFold2 to predict protein structure at unprecedented levels of accuracy (Jumper et al. 2021). Nevertheless, for almost no other task is EI as crucial as for SAV effect prediction (Bromberg and Rost 2007). Although different sources of input information matter, when MSAs are available, they trump all other features (Hecht et al. 2015). Even models building on the simplest EI, e.g., the BLOSUM62 matrix condensing bio-physical constraints into a 20 × 20 substitution matrix (Ng and Henikoff 2003) with no distinction between E481K (amino acid E at residue position 481 mutated to amino acid K) and E484K (part of SARS-CoV-2 Delta variant), or a simple conservation weight (Reeb et al. 2020) with no distinction of D484Q and D484K, almost reach the performance of much more complex and seemingly *advanced* methods.

**Fig. 1:**
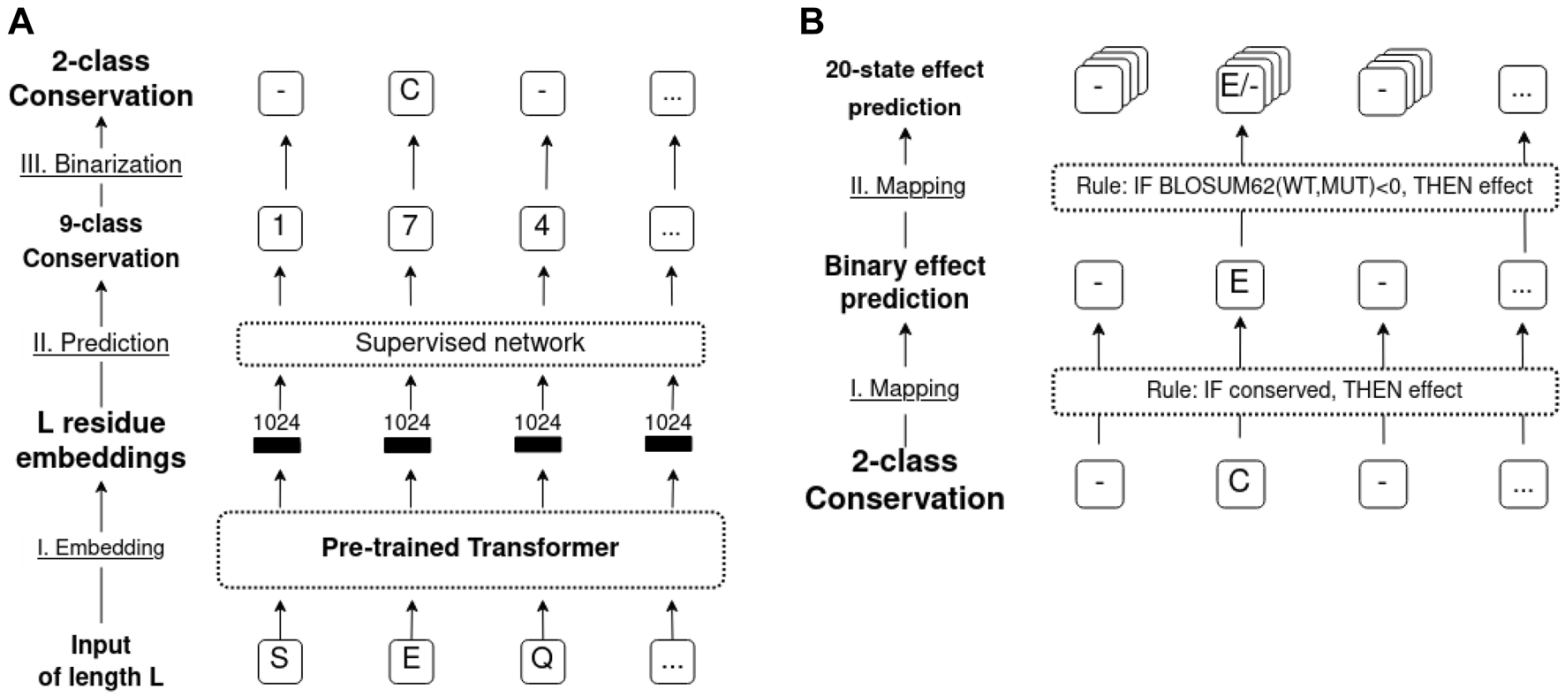
Sketch of methods. Panel A sketches the conservation prediction pipeline: (I) embed protein sequence (“SEQ”) using a pLM [here: ProtBERT, ProtT5 (Elnaggar et al. 2021) or ESM-1b (Meier et al. 2021)]. (II) Input embedding into supervised method (here: logistic regression, FNN or CNN) to predict conservation in 9-classes as defined by ConSurf-DB (Ben Chorin et al. 2020). (III) Map nine-class predictions > 5 to *conserved* (C), others to *non-conserved* (−). Panel B shows the use of binary conservation predictions as input to SAV effect prediction by (I) considering all residue positions predicted as conserved (C) as effect (E), all others as neutral (ProtT5cons-19equal and ConSeq-19equal). (II) Residues predicted as conserved are further split into specific substitutions (SAVs) predicted to have an effect (E) or not (−) if the corresponding BLOSUM62 score is < 0, all others are predicted as neutral (ProtT5-beff, ConSeq-BLOSUM62)

**Embeddings capture language of life written in proteins.** Every year, algorithms improve natural language processing (NLP), in particular by feeding large text corpora into Deep Learning (DL)-based Language Models (LMs). These advances have been transferred to protein sequences by learning to predict masked or missing amino acids using large databases of raw protein sequences as input (Alley et al. 2019; Bepler and Berger 2019a, 2021; Elnaggar et al. 2021; Heinzinger et al. 2019; Madani et al. 2020; Ofer et al. 2021; Rao et al. 2020; Rives et al. 2021). Processing the information learned by such protein LMs (pLMs), e.g., by constructing 1024-dimensional vectors of the last hidden layers, yields a representation of protein sequences referred to as embeddings [Fig. 1 in (Elnaggar et al. 2021)]. Embeddings have succeeded as exclusive input to predicting secondary structure and subcellular location at performance levels almost reaching (Alley et al. 2019; Heinzinger et al. 2019; Rives et al. 2021) or even exceeding (Elnaggar et al. 2021; Littmann et al. 2021c; Stärk et al. 2021) state-of-the-art (SOTA) methods using EI from MSAs as input. Embeddings even succeed in substituting sequence similarity for homology-based annotation transfer (Littmann et al. 2021a, b) and in predicting the effect of mutations on protein–protein interactions (Zhou et al. 2020). The power of such embeddings has been increasing with the advance of algorithms (Bepler and Berger 2021; Elnaggar et al. 2021; Rives et al. 2021). ESM-1v demonstrated pre-trained pLMs predicting SAV effect without any supervision at state-of-the-art level on DMS data using solely mask reconstruction probabilities (Meier et al. 2021). Naturally, there will be some limit to such improvements. However, the advances over the last months prove that this limit had not been reached by the end of 2020.

Here, we analyzed ways of using embeddings from pre-trained pLMs to predict the effect of SAVs upon protein function with a focus on molecular function, using experimental data from DMS (Esposito et al. 2019) and PMD (Kawabata et al. 1999). The embeddings from the pre-trained pLMs were not altered or optimized to suit the subsequent 2^nd^ step of supervised training on data sets with more limited annotations. In particular, we assessed two separate supervised prediction tasks: conservation and SAV effects. First, we utilized pre-trained pLMs (ProtBert, ProtT5, ESM-1b) as static feature encoders (without fine-tuning the pLMs) to derive input embeddings for developing a method predicting the conservation that we could read off a family of aligned sequences (MSA) for each residue without actually generating the MSA. Second, we trained a Logistic Regression (LR) ensemble to predict SAV effect using (2a) the predictions of the best conservation predictor (ProtT5cons) together with (2b) substitution scores of BLOSUM62 and (2c) substitution probabilities of the pLM ProtT5. While substitution probabilities alone already correlated with DMS scores, we observed that adding conservation predictions together with BLOSUM62 increased performance. The resulting model for Variant Effect Score Prediction without Alignments (VESPA) was competitive with more complex solutions in terms of correlation with experimental DMS scores and computational and environmental costs. Additionally, for a small drop in prediction performance, we created a computationally more efficient method, dubbed VESPA-light (or short: VESPAl), by excluding substitution probabilities to allow proteome-wide analysis to complete after the coffee break on a single machine (40 min for human proteome on one Nvidia Quadro RTX 8000).

## Methods

### Data sets

In total, we used five different datasets. *ConSurf10k* was used to train and evaluate a model on residue conservation prediction. *Eff10k* was used to train SAV effect prediction. *PMD4k* and *DMS4* were used as test sets to assess the prediction of binary SAV effects. The prediction of continuous effect scores was evaluated on *DMS39*.

***ConSurf10k*** **assessed conservation.** The method predicting residue conservation used *ConSurf-DB* (Ben Chorin et al. 2020). This resource provided sequences and conservation for 89,673 proteins. For all, experimental high-resolution three-dimensional (3D) structures were available in the Protein Data Bank (PDB) (Berman et al. 2000). As standard-of-truth for the conservation prediction, we used the values from ConSurf-DB generated using HMMER (Mistry et al. 2013), CD-HIT (Fu et al. 2012), and MAFFT-LINSi (Katoh and Standley 2013) to align proteins in the PDB (Burley et al. 2019). For proteins from families with over 50 proteins in the resulting MSA, an evolutionary rate at each residue position is computed and used along with the MSA to reconstruct a phylogenetic tree. The ConSurf-DB conservation scores ranged from 1 (most variable) to 9 (most conserved). The PISCES server (Wang and Dunbrack 2003) was used to redundancy reduce the data set, such that no pair of proteins had more than 25% pairwise sequence identity. We removed proteins with resolutions > 2.5 Å, those shorter than 40 residues, and those longer than 10,000 residues. The resulting data set (ConSurf10k) with 10,507 proteins (or domains) was randomly partitioned into training (9392 sequences), cross-training/validation (555), and test (519) sets.

***Eff10k*** **assessed SAV effects.** This dataset was taken from the SNAP2 development set (Hecht et al. 2015). It contained 100,737 binary SAV-effect annotations (neutral: 39,700, effect: 61,037) from 9594 proteins. The set was used to train an ensemble method for SAV effect prediction. For this, we replicated the cross-validation (CV) splits used to develop SNAP2 by enforcing that clusters of sequence-similar proteins were put into the same CV split. More specifically, we clustered all sequence-similar proteins (PSI-BLAST *E* value < 1e-3) using single-linkage clustering, i.e., all connected nodes (proteins) were put into the same cluster. By placing all proteins within one cluster into the same CV split and rotating the splits, such that every split was used exactly once for testing, we ascertained that no pair of proteins between train and test shared significant sequence similarity (PIDE). More details on the dataset are given in SNAP2 (Hecht et al. 2015).

***PMD4k*** **assessed binary SAV effects.** From Eff10k, we extracted annotations that were originally adopted from PMD (“no change” as “neutral”; annotations with any level of increase or decrease in function as “effect”). This yielded 51,817 binary annotated SAVs (neutral: 13,638, effect: 38,179) in 4061 proteins. PMD4k was exclusively used for testing. While these annotations were part of Eff10k, all performance estimates for PMD4k were reported only for the PMD annotations in the testing subsets of the cross-validation splits. As every protein in Eff10k (and PMD4k) was used exactly once for testing, we could ascertain that there was no significant (prediction by homology-based inference possible) sequence-similarity between PMD4k and our training splits.

***DMS4*** **sampled large-scale DMS in vitro experiments annotating binary SAV effects.** This set contained binary classifications (effect/non-effect) for four human proteins (corresponding genes: BRAC1, PTEN, TPMT, PPARG) generated previously (Reeb 2020). These were selected as they were the first proteins with comprehensive DMS experiments including synonymous variants (needed to map from continuous effect scores to binary effect vs. neutral) resulting in 15,621 SAV annotations (Findlay et al. 2018; Majithia et al. 2016; Matreyek et al. 2018). SAVs with beneficial effect (= gain of function) were excluded, because they disagree between experiments (Reeb et al. 2020). The continuous effect scores of the four DMS experiments were mapped to binary values (effect/neutral) by considering the 95% interval around the mean of all experimental measurements as neutral, and the 5% tails of the distribution as “effect”, as described in more detail elsewhere (Reeb et al. 2020). In total, the set had 11,788 neutral SAVs and 3516 deleterious effect SAVs. Additionally, we used two other thresholds: the 90% interval from mean (8926 neutral vs. 4545 effect) and the 99% interval from mean (13,506 neutral vs. 1,548 SAVs effect).

***DMS39*** **collected DMS experiments annotating continuous SAV effects.** This set was used to assess whether the methods introduced here, although trained only on binary effect data from Eff10k, had captured continuous effect scales as measured by DMS. The set was a subset of 43 DMS experiments assembled for the development of DeepSequence (Riesselman et al. 2018). From the original compilation, we excluded an experiment on tRNA as it is not a protein, on the toxin–antitoxin complex as it comprises multiple proteins and removed experiments for which only double variants existed. DMS39 contained 135,665 SAV scores, in total. The number of SAVs per experiment varied substantially between the 39 with an average of 3625 SAVs/experiment, a median of 1962, a minimum of 21, and a maximum of 12,729. However, to avoid any additional biases in the comparison to other methods, we avoided any further filtering step.

### Input features

For the prediction of residue conservation, all newly developed methods exclusively trained on embeddings from pre-trained pLMs without fine-tuning those (no gradient was backpropagated to the pLM). The predictions of the best-performing method for conservation prediction were used in a second step together with substitution scores from BLOSUM62 and substitution probabilities from ProtT5 as input features to predict binary SAV effects.

**Embeddings from pLMs:** For conservation prediction, we used embeddings from the following pLMs: *ProtBert* (Elnaggar et al. 2021) based on the NLP (Natural Language Processing) algorithm BERT (Devlin et al. 2019) trained on Big Fantastic Database (BFD) with over 2.3 million protein sequences (Steinegger and Söding 2018), ESM-1b (Rives et al. 2021) that is conceptually similar to (Prot)BERT (both use a Transformer encoder) but trained on UniRef50 (The UniProt Consortium 2021) and *ProtT5-XL-U50* (Elnaggar et al. 2021) (for simplicity referred to as *ProtT5*) based on the NLP sequence-to-sequence model T5 (transformer encoder–decoder architecture) (Raffel et al. 2020) trained on BFD and fine-tuned on Uniref50. All embeddings were obtained from the bio_embeddings pipeline (Dallago et al. 2021). As described in ProtTrans, only the encoder side of ProtT5 was used and embeddings were extracted in half-precision (Elnaggar et al. 2021). The per-residue embeddings were extracted from the last hidden layer of the models with size 1024 × L (1280 for ESM-1b), where L is the length of the protein sequence and 1024 (or 1280 for ESM-1b) is the dimension of the hidden states/embedding space of ESM-1b, ProtBert, and ProtT5.

**Context-dependent substitution probabilities:** The training objective of most pLMs is to reconstruct corrupted amino acids from their non-corrupted protein sequence context. Repeating this task on billions of sequences allows pLMs to learn a probability of how likely it is to observe a token (an amino acid) at a certain position in the protein sequence. After pre-training, those probabilities can be extracted from pLMs by masking/corrupting one token/amino acid at a time, letting the model reconstruct it based on non-corrupted sequence context and repeating this for each token/amino acid in the sequence. For each protein, this gives a vector of length L by 20 with L being the protein’s length and 20 being the probability distribution over the 20 standard amino acids. It was shown recently (Meier et al. 2021) that these probabilities provide a context-aware estimate for the effect of SAVs, i.e., the reconstruction probabilities depend on the protein sequence, and other methods have made use of similar probabilities (Hopf et al. 2017; Riesselman et al. 2018). To generate input features for our SAV effect predictor, we used, as suggested by Meier et al. (2021), the log-odds ratio between the probability of observing the wild-type amino acid at a certain position and the probability of observing a specific mutant at the same position: $\mathrm{log}\left(p\left({X}_{i,mutant}\right)\right)-\mathrm{log}(p\left({X}_{i,wildtype}\right))$. The term $p\left({X}_{i,mutant}\right)$ described the probability of an SAV occurring at position *i* and $p\left({X}_{i,wildtype}\right)$ described the corresponding probability of the wild-type occurrence (native amino acid). To extract these probabilities for SAV effect prediction, we only considered the pLM embeddings correlating best with conservation (ProtT5). Additionally, we extracted probabilities for ProtBert on ConSurf10k to analyze in more detail the mistakes that ProtBert makes during reconstruction (SOM Fig. S5, S6).

**Context-independent BLOSUM62 substitution scores:** The BLOSUM substitution matrix gives a log-odds ratio for observing an amino acid substitution irrespective of its position in the protein (Henikoff and Henikoff 1992), i.e., the substitution score will not depend on a specific protein or the position of a residue within a protein but rather focuses on bio-chemical and bio-physical properties of amino acids. Substitution scores in BLOSUM were derived from comparing the log-odds of amino acid substitutions among well-conserved protein families. Typically applied to align proteins, BLOSUM scores are also predictive of SAV effects (Ng and Henikoff 2003; Sruthi et al. 2020).

### Method development

In our three-stage development, we first compared different combinations of network architectures and pLM embeddings to predict residue conservation. Next, we combined the best conservation prediction method with BLOSUM62 substitution scores to develop a simple rule-based prediction of binary SAV effects. In the third step, we combined the predicted conservation, BLOSUM62, and substitution probabilities to train a new method predicting SAV effects for binary data from Eff10k and applied this method to non-binary DMS data.

**Conservation prediction** (ProtT5cons, Fig. 1A): Using either ESM-1b, ProtBert, or ProtT5 embeddings as input (Fig. 1a), we trained three supervised classifiers to distinguish between nine *conservation classes* taken from ConSurf-DB (early stop when optimum reached for ConSurf10k validation set). The objective of this task was to learn the prediction of family conservation from ConSurf-DB (Ben Chorin et al. 2020) based on the nine conservation classes introduced by that method that range from 1 (variable) to 9 (conserved) for each residue in a protein, i.e., this task implied a multi-class per-residue prediction. Cross-entropy loss together with Adam (Kingma and Ba 2014) was used to optimize each network toward predicting one out of nine conservation classes for each residue in a protein (per-token/per-residue task).

The models were: (1) standard Logistic Regression (LR) with 9000 (9 k) free parameters; (2) feed-forward neural network (FNN; with two FNN layers connected through the so-called ReLU (rectified linear unit) activations (Fukushima 1969); dropout rate 0.25; 33 k free parameters); (3) standard convolutional neural network (CNN; with two convolutional layers with a window size of 7, connected through ReLU activations; dropout rate of 0.25; 231 k free parameters). To put the number of free parameters into perspective: the ConSurf10k data set contained about 2.7 million samples, i.e., an order of magnitude more samples than free parameters of the largest model. On top of the 9-class prediction, we implemented a binary classifier (*conserved*/*non-conserved*; threshold for projecting nine to two classes optimized on validation set). The best-performing model (CNN trained on ProtT5) was referred to as ProtT5cons.

**Rule-based binary SAV effect prediction** (ProtT5beff, Fig. 1B): For rule-based binary SAV effect (*effect/neutral*) prediction, we considered multiple approaches. The first and simplest approach was to introduce a threshold to the output of ProtT5cons (no optimization on SAV data). Here, we marked all residues predicted to be conserved (conservation score > 5) as “effect”; all others as “neutral”. This first level treated all 19 non-native SAVs at one sequence position equally (referred to as “19equal” in tables and figures). To refine, we followed the lead of SIFT (Ng and Henikoff 2003) using the BLOSUM62 (Henikoff and Henikoff 1992) substitution scores. This led to the second rule-based method dubbed *BLOSUM62bin* which can be considered a naïve baseline: SAVs less likely than expected (negative values in BLOSUM62) were classified as “effect”; all others as “neutral”. Next, we combined both rule-based classifiers to the third rule-based method, dubbed *ProtT5beff* (*“*effect” if ProtT5cons predicts conserved, i.e., value > 5, and BLOSUM62 negative, otherwise “neutral”, Fig. 1b). This method predicted binary classifications (effect/neutral) of SAVs without using any experimental data on SAV effects for optimization by merging position-aware information from ProtT5cons and variant-aware information from BLOSUM62.

**Supervised prediction of SAV effect scores** (*VESPA* and *VESPAl)*: For variant effect score prediction without alignments (VESPA), we trained a balanced logistic regression (LR) ensemble method as implemented in SciKit (Pedregosa et al. 2011) on the cross-validation splits of Eff10k. We rotated the ten splits of Eff10k, such that each data split was used exactly once for testing, while all remaining splits were used for training. This resulted in ten individual LRs trained on separate datasets. All of those were forced to share the same hyper-parameters. The hyper-parameters that differed from SciKit’s defaults were: (1) *balanced weights*: class weights were inversely proportional to class frequency in input data; (2) *maximum number of iterations taken for the solvers to converge* was set to 600. The learning objective of each was to predict the probability of binary class membership (effect/neutral). By averaging their output, we combined the ten LRs to an ensemble method: $VESPA=ensemble \,of\, LRs=\frac{1}{10}\sum_{i=1}^{10}L{R}_{i}$. The output of VESPA is bound to [0,1] and by introducing a threshold can be readily interpreted as a probability for an SAV to be “neutral” (VESPA < 0.5) or to have “effect” (VESPA ≥ 0.5). As input for VESPA, we used 11 features to derive one score for each SAV; nine were the position-specific conservation probabilities predicted by ProtT5cons; one was the variant-specific substitution score from BLOSUM62, the other the variant- and position-specific log-odds ratio of ProtT5’s substitution probabilities. To reduce the computational costs of VESPA, we introduced the “light” version VESPAl using only conservation probabilities and BLOSUM62 as input and thereby circumventing the computationally more costly extraction of the log-odds ratio. Both VESPA and VESPAl were only optimized on binary effect data from Eff10k and never encountered continuous effect scores from DMS experiments during any optimization.

### Evaluation

**Conservation prediction—ProtT5cons:** To put the performance of ProtT5cons into perspective, we generated ConSeq (Berezin et al. 2004) estimates for conservation through PredictProtein (Bernhofer et al. 2021) using MMseqs2 (Steinegger and Söding 2018) and PSI-BLAST (Altschul et al. 1997) to generate MSAs. These were “estimates” as opposed to the standard-of-truth from ConSurf-DB, because, although they actually generated entire MSAs, the method for MSA generation was “just” MMseqs2 as opposed to HMMER (Mistry et al. 2013), and MAFFT-LINSi (Katoh and Standley 2013) for ConSurf-DB and the computation of weights from the MSA also required less computing resources. A random baseline resulted from randomly shuffling ConSurf-DB values.

**Binary effect prediction—ProtT5beff:** To analyze the performance of VESPA and VESPAl, we compared results to SNAP2 (Hecht et al. 2015) at the default binary threshold (score > − 0.05, default value suggested in original publication) on PMD4k and DMS4. Furthermore, we evaluated the rule-based binary SAV effect prediction ProtT5beff on the same datasets. To assess to which extent performance of ProtT5beff could be attributed to mistakes in ProtT5cons, we replaced residue conservation from ProtT5cons with conservation scores from ConSeq and applied the same two rule-based approaches as explained above (*ConSeq 19equal*: conserved predictions at one sequence position were considered “effect” for all 19 non-native SAVs and *ConSeq blosum62*: only negative BLOSUM62 scores at residues predicted as conserved were considered “effect”; all others considered “neutral” with both using the same threshold in conservation as for our method, i.e., conservation > 5 for effect) for PMD4k and DMS4. This failed for 122 proteins on PMD4k (3% of PMD4k), because the MSAs were deemed too small. We also compared ProtT5beff to the baseline based only on BLOSUM62 with the same thresholds as above (BLOSUM62bin). Furthermore, we compared to SNAP2 at default binary threshold of effect: SNAP2 score > − 0.05 (default value suggested in original publication). SNAP2 failed for four of the PMD4k proteins (0.1% of PMD4k). For the random baseline, we randomly shuffled ground truth values for each PMD4k and DMS4.

**Continuous effect prediction—VESPA:** We evaluated the performance of VESPA and VESPAl on DMS39 comparing to MSA-based DeepSequence (Riesselman et al. 2018) and GEMME (Laine et al. 2019), and the pLM-based ESM-1v (Meier et al. 2021). Furthermore, we evaluated log-odds ratios from ProtT5’s substitution probabilities and BLOSUM62 substitution scores as a baseline. The DeepSequence predictions were copied from the supplement to the original publication (Riesselman et al. 2018), GEMME correlation coefficients were provided by the authors, and ESM-1v predictions were replicated using the online repository of ESM-1v. We used the publicly available ESM-1v scripts to retrieve “*masked-marginals*” for each of the five ESM-1v models and averaged over their outputs, because this strategy gave best performance according to the authors. If a protein was longer than 1022 (the maximum sequence length that ESM-1v can process), we split the sequence into non-overlapping chunks of length 1022. VESPA, VESPAl, and ESM-1v predictions did not use MSAs and therefore provided results for the entire input sequences, while DeepSequence and GEMME were limited to residues to which enough other protein residues were aligned in the MSAs.

**Performance measures:** We applied the following standard performance measures:

1 $${\text{Q2 = 100}} \cdot \frac{{\text{(Number of residues predicted correctly in 2 states)}}}{{\text{(Number of all residues)}}}.$$

Q2 scores (Eq. 1) described both binary predictions (conservation and SAV effect). The same held for F1-scores (Eq. 6, 7) and MCC (Matthews Correlation Coefficient, Eq. 8). We defined conserved/effect as the positive class and non-conserved/neutral as the negative class (indices “ + ” for positive, “−“ for negative) and used the standard abbreviations of TP (true positives: number of residues predicted and observed as conserved/effect), TN (true negatives: predicted and observed as non-conserved/neutral), FP (false positives: predicted conserved/effect, observed non-conserved/neutral), and FN (false negatives: predicted non-conserved/neutral, observed conserved/effect)

2 $$Accurac{y}_{+}=Precisio{n}_{+}=Positive \,Predicted\, Value=\frac{TP}{TP+FP}$$

3 $$Accurac{y}_{-}=Precisio{n}_{-}=Negative Predicted Value=\frac{TN}{TN+FN}$$

4 $${Coverage}_{+}=Reca{ll}_{+}=Sensitivity=\frac{TP}{TP+FN}$$

5 $$Coverage\_=Recal{l}_{-}=Specificity=\frac{TN}{TN+FP}$$

6 $$F{1}_{+}=100 \bullet 2\bullet \frac{Precisio{n}_{+} \bullet Recal{l}_{+}}{Precisio{n}_{+} + Recal{l}_{+}}$$

7 $$F{1}_{-}=100\bullet 2\bullet \frac{Precisio{n}_{-} \bullet Recal{l}_{-}}{Precisio{n}_{-} + Recal{l}_{-}}$$

8 $$MCC=\frac{TP\bullet TN-FP\bullet FN}{\sqrt{(TP+FP)\bullet (TP+FN)\bullet (TN+FP)\bullet (TN+FN)}}$$

9 $$Q9=100\bullet \frac{Number \,of\, residues\, predicted\,correctly \,in \,9\, states}{Number\, of\, all \,residues}.$$

Q9 is exclusively used to measure performance for the prediction of nine classes of conservation taken from ConSurf-DB. Furthermore, we considered the Pearson correlation coefficient

10 $${{r}_{P}=\rho }_{X,Y}=\frac{cov(X,Y)}{{\sigma }_{X}{\sigma }_{Y}},$$

and the Spearman correlation coefficient where raw scores (X, Y of Eq. 10) are converted to ranks

11 $${r}_{S}={\rho }_{r{g}_{X},r{g}_{Y}}=\frac{cov(r{g}_{X},r{g}_{Y})}{{\sigma }_{Xr{g}_{X}}{\sigma }_{r{g}_{Y}}}$$

for continuous effect prediction.

**Error estimates:** We estimated symmetric 95% confidence intervals (CI Eq. 12) for all metrics using bootstrapping (Efron et al. 1996) by computing 1.96* standard deviation (SD) of randomly selected variants from all test sets with replacement over *n* = 1000 bootstrap sets

12 $$CI=1.96\bullet SD=1.96\bullet \sqrt{\frac{{\sum ({y}_{i}-\overline{y })}^{2} }{n},}$$

with ${y}_{i}$ being the metric for each bootstrap sample and $\overline{y }$ the mean over all bootstrap samples. We considered differences in performance significant if two CIs did not overlap.

Probability entropy: To investigate the correlation between embeddings and conservation classes of ConSurf-DB, we computed the entropy of pLM substitution probabilities (*p*) as

13 $$Entropy ({p}_{1},\dots ,{p}_{n})=-\sum_{i=1}^{n}{p}_{i}{\mathrm{log}}_{2}{p}_{i}.$$

## Results

We first showed that probabilities derived from pLMs sufficed for the prediction of residue conservation from pLM embeddings without using MSAs (data set *ConSurf10k*; method *ProtT5cons*). Next, we presented a non-parametric rule-based SAV effect prediction based on predicted conservation (IF “predicted conserved” THEN “predict effect”; method *ProtT5beff*). We refined the rule-based system through logistic regression (LR) to predict SAV effect on variants labeled with “effect” or “neutral” (data set *Eff10k*; methods *VESPA, VESPAl*). Finally, we established that these new methods trained on binary data (effect/neutral) from *Eff10k* correlated with continuous DMS experiments.

**Fig. 2:**
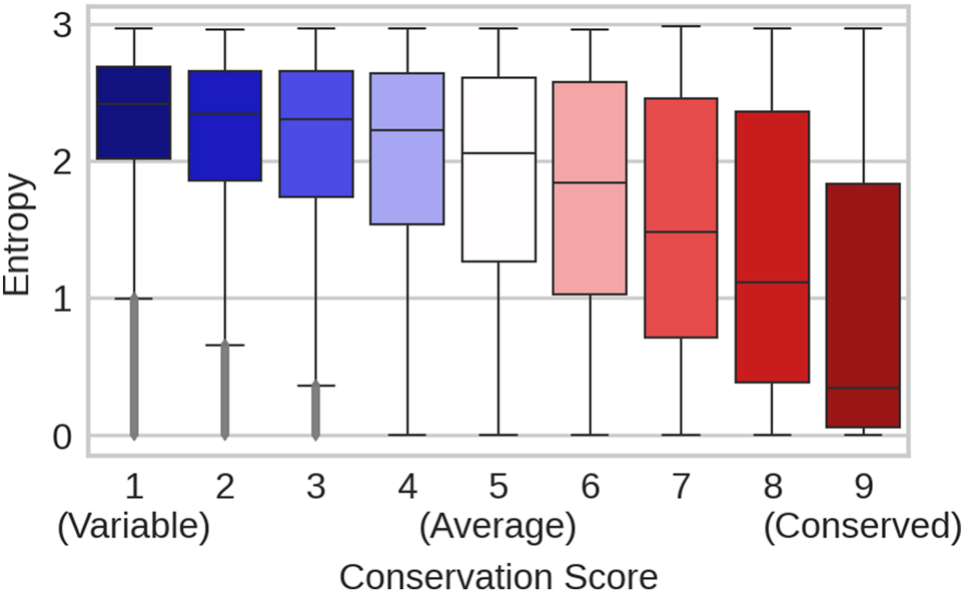
pLMs captured conservation without supervised training or MSAs. ProtBert was optimized to reconstruct corrupted input tokens from non-corrupted sequence context (masked language modeling). Here, we corrupted and reconstructed all proteins in the ConSurf10k dataset, one residue at a time. For each residue position, ProtBert returned the probability for observing each of the 20 amino acids at that position. The higher one probability (and the lower the corresponding entropy), the more certain the pLM predicts the corresponding amino acid at this position from non-corrupted sequence context. Within the displayed boxplots, medians are depicted as black horizontal bars; whiskers are drawn at the 1.5 interquartile range. The *x*-axis gives categorical conservation scores (1: highly variable, 9: highly conserved) computed by ConSurf-DB (Ben Chorin et al. 2020) from multiple sequence alignments (MSAs); the *y*-axis gives the probability entropy (Eq. 13) computed without MSAs. The two were inversely proportional with a Spearman’s correlation of -0.374 (Eq. 11), i.e., the more certain ProtBert’s prediction, the lower the entropy and the higher the conservation for a certain residue position. Apparently, ProtBert had extracted information correlated with residue conservation during pre-training without having ever seen MSAs or any labeled data

**Embeddings predicted conservation:** First, we established that protein Language Models (pLMs) capture information correlated with residue conservation without ever seeing any such labels. As a standard-of-truth, we extracted the categorical conservation scores ranging from 1 to 9 (9: highly conserved, 1: highly variable) from ConSurf-DB (Ben Chorin et al. 2020) for a non-redundant subset of proteins with experimentally known structures (data set ConSurf10k). Those conservation scores correlated with the mask reconstruction probabilities output by ProtBert (Fig. 2). More specifically, one amino acid was corrupted at a time and ProtBert reconstructed it from non-corrupted sequence context. For instance, when corrupting and reconstructing all residues in ConSurf10k (one residue at a time), ProtBert assigned a probability to the native and to each of the 19 non-native (SAVs) amino acids for each position in the protein. Using those “*substitution probabilities*”, ProtBert correctly predicted the native amino acid in 45.3% of all cases compared to 9.4% for a random prediction of the most frequent amino acid (Fig. S4). The entropy of these probability distributions correlated slightly with conservation (Fig. 2, Spearman’s R = -−0.374) although never trained on such labels.

**Fig. 3:**
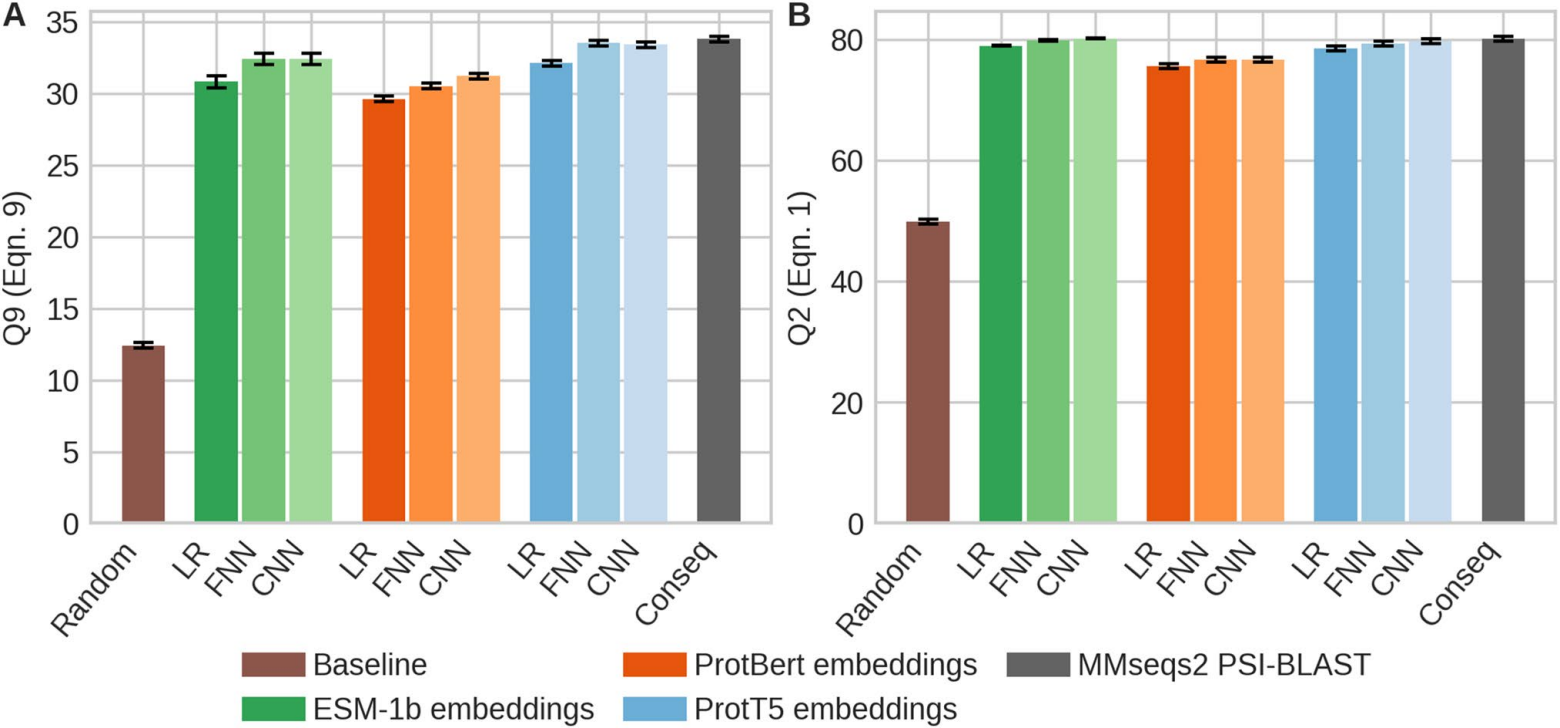
Conservation predicted accurately from embeddings. Data: hold-out test set of *ConSurf10k* (519 sequences); panel A: nine-state per-residue accuracy (Q9, Eq. 9) in predicting conservation as defined by ConSurf-DB (Ben Chorin et al. 2020); panel B: two-state per-residue accuracy (Q2, Eq. 1; conservation score > 5: conserved, non-conserved otherwise). Supervised models (trained on *ConSurf10k*): **LR**: logistic regression (9,000 = 9 k free parameters), *FNN* feed-forward network (33 k parameters), and *CNN* convolutional neural network (231 k parameters with 0.25 dropout rate); methods: *ConSeq* computation of conservation weight through multiple sequence alignments (MSAs) (Berezin et al. 2004); *Random* random label swap. Model inputs were differentiated by color (green: ESM-1b embeddings (Rives et al. 2021), red: ProtBert embeddings (Elnaggar et al. 2021), blue: ProtT5 embeddings (Elnaggar et al. 2021), gray: MSAs (MMseqs2 (Steinegger and Söding 2017), and PSI-BLAST (Altschul et al. 1997)). Black whiskers mark the 95% confidence interval (± 1.96 SD; Eq. 12). ESM-1b and ProtT5 embeddings outperformed those from ProtBERT (Elnaggar et al. 2021); differences between ESM-1b and ProtT5 were not statistically significant, but ProtT5 consistently outperformed ESM-1b in all metrics but Q2 (Table S1). ESM-1b and ProtT5 as input to the CNN came closest to ConSeq (Table S1)

Next, we established that residue conservation can be predicted directly from embeddings by training a supervised network on data from ConSurf-DB. We exclusively used embeddings of pre-trained pLMs (ProtT5, ProtBert (Elnaggar et al. 2021), ESM-1b (Rives et al. 2021)), as input to relatively simple machine learning models (Fig. 1). Even the simplistic logistic regression (LR) reached levels of performance within about 20% of ConSeq (Berezin et al. 2004) conservation scores, which were derived from MSAs generated by the fast alignment method MMseqs2 (Steinegger and Söding 2017) (Fig. 3). The top prediction used ProtT5 embeddings which consistently outperformed predictions from ESM-1b and ProtBERT embeddings. For all three types of embeddings, the CNN outperformed the FNN, and these outperformed the LR. Differences between ProtBert and ProtT5 were statistically significant (at the 95% confidence interval, Eq. 12), while improvements from ProtT5 over ESM-1b were mostly insignificant. The ranking of the embeddings and models remained stable across several performance measures (F1_effect_, F1_neutral_, MCC, Pearson correlation coefficient, Table S1).

ConSurf-DB (Ben Chorin et al. 2020) simplifies family conservation to a single digit integer (9: highly conserved, 1: highly variable). We further reduced these classes to a binary classification (conserved/non-conserved) to later transfer information from conservation to binary SAV effect (effect/neutral) more readily. The optimal threshold for a binary conservation prediction was 5 (> 5 conserved, Fig. S1). However, performance was stable across a wide range of choices: between values from 4 to 7, MCC (Eq. 8) changed between 0.60 and 0.58, i.e., performance varied by 3.3% for 44.4% of all possible thresholds (Fig. S1). This was explained by the nine- and two-class confusion matrices (Fig. S2 and S3) for *ProtT5cons*, which showed that most mistakes were made between neighboring classes of similar conservation, or between the least conserved classes 1 and 2.

**Fig. 4:**
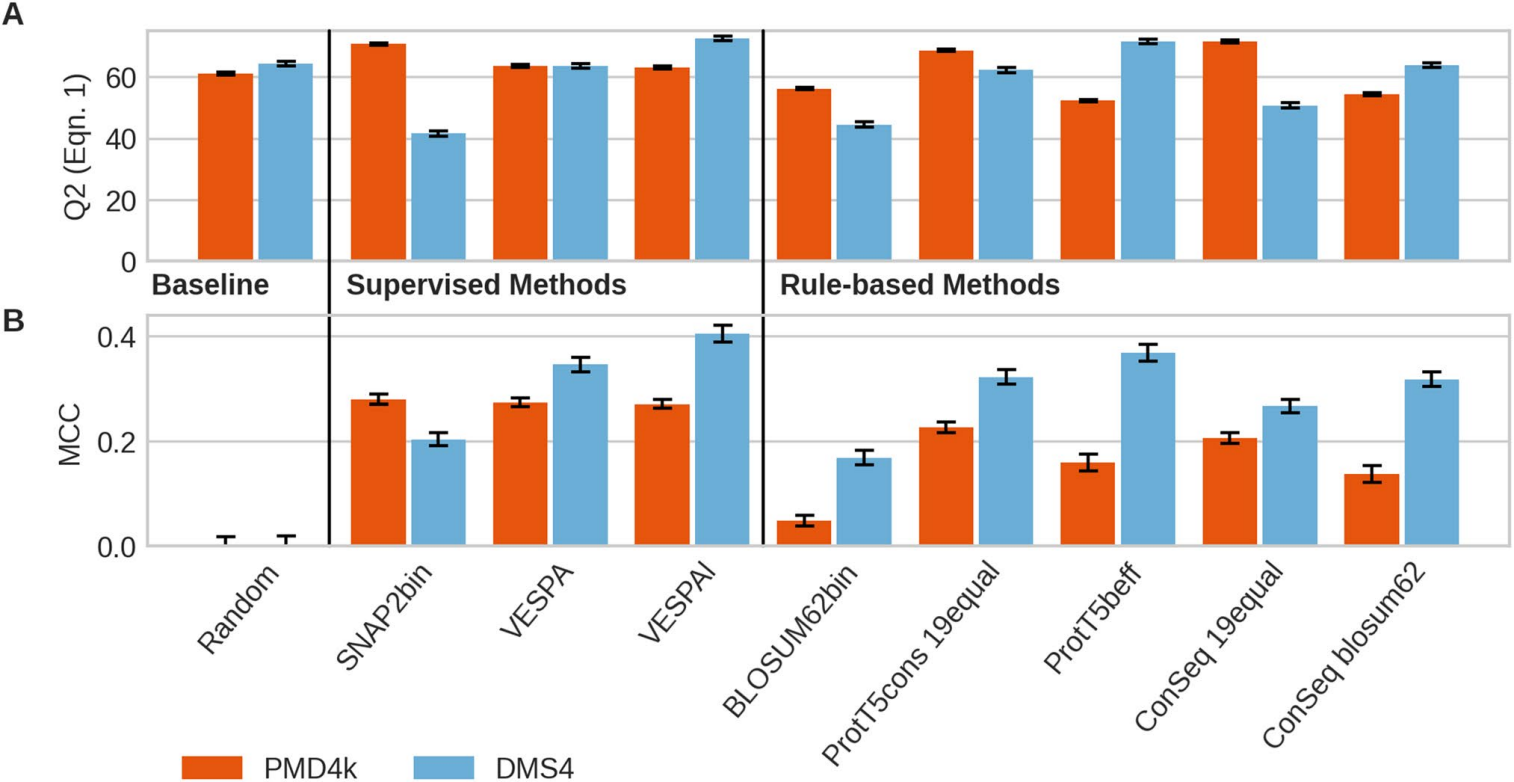
Embedding-based binary SAV effect prediction is seemingly competitive. Data: *PMD4k* (red bars; 4 k proteins from PMD (Kawabata et al. 1999)); *DMS4* (blue bars) first four human proteins (BRAC1, PTEN, TPMT, PPARG) with comprehensive experimental DMS measurements including synonyms (here 95% threshold) (Reeb et al. 2020). Methods: SUPERVISED: **a** *SNAP2bin*: effect SNAP2 score > **− **0.05, otherwise neutral; **b** *VESPA*: effect VESPA score >  = 0.5, otherwise neutral; **c** *VESPAl*: effect VESPAl score >  = 0.5, otherwise neutral. RULE-BASED: **d** *BLOSUM62bin*: irrespective of residue position, negative BLOSUM62 scores predicted as effect, others as neutral; **e** *ProtT5cons*|*ConSeq 19equal*: all 19 non-native SAVs predicted equally: effect if ProtT5cons|ConSeq predicted residue position to be conserved, otherwise neutral; **f** *ProtT5beff*|*ConSeq blosum62*: effect if ProtT5cons|ConSeq predicts conserved and BLOSUM62 negative, otherwise neutral. BASELINE: **g** *Random*: random shuffle of experimental labels. All values for DMS4 computed for binary (effect/neutral) mapping of experimental DMS values with panel A giving the two-state per-residue accuracy (Q2, Eq. 1) and panel B giving the Matthews Correlation Coefficient (MCC, Eq. 8). Error bars: Black bars mark the 95% confidence interval (± 1.96 SD, Eq. 12). For all methods, the MCC differences between the two data sets PMD4k and DMS4 were statistically significant (exception: random)

**Conservation-based prediction of binary SAV effect better for DMS4 than for PMD4k?** Next, we established that we could use the predicted conservation of ProtT5cons for rule-based binary SAV effect prediction without any further optimization and without any MSA. In using predicted conservation to proxy SAV effect, we chose the method best in conservation prediction, namely the CNN using ProtT5 embeddings (method dubbed *ProtT5cons*, Fig. 1B). The over-simplistic approach of considering any residue predicted as conserved to have an effect irrespective of the SAV (meaning: treat all 19 non-native SAVs alike) was referred to as “19equal”. We refined this rule-based approach by combining conservation prediction with a binary BLOSUM62 score (effect: if ProtT5cons predicted conserved AND BLOSUM62 < 0, neutral otherwise), which we referred to as *ProtT5beff*. For PMD4k, the following results were common to all measures reflecting aspects of precision and recall through a single number (F1_effect_, F1_neutral_ and MCC). First, the expert method SNAP2 trained on Eff10k (superset of PMD4k) achieved numerically higher values than all rule-based methods introduced here. Second, using the same SAV effect prediction for all 19 non-native SAVs consistently reached higher values than using the BLOSUM62 values (Fig. 4 and Table 1: *19equal* higher than *blosum62*). For some measures (Q2, F1_effect_), values obtained using ConSeq for conservation (i.e., a method using MSAs) were higher than those for the ProtT5cons prediction (without using MSAs), while for others (MCC, F1_neutral_**),** this was reversed (Fig. 4, Table 1, Table S2).

**Table 1:** Performance in binary SAV effect prediction^a^

Data set	PMD4k		DMS4	
Method/metric	Q2 (Eq. 1)	MCC (Eq. 8)	Q2 (Eq. 1)	MCC (Eq. 8)
Random	61.08% ± 0.41	**−** 0.002 ± 0.016	64.27% ± 0.76	**−** 0.001 ± 0.018
Supervised methods				
*SNAP2bin*	70.66% ± 0.39	0.280 ± 0.010	41.55% ± 0.82	0.204 ± 0.012
*VESPA*	63.52% ± 0.43	0.274 ± 0.086	63.56% ± 0.79	0.346 ± 0.014
*VESPAl*	63.04% ± 0.43	0.271 ± 0.085	72.59% ± 0.72	**0.405 ± 0.016**
Rule-based methods				
*BLOSUM62bin*	56.17% ± 0.43	0.049 ± 0.010	44.47% ± 0.84	0.169 ± 0.014
*ProtT5cons-19equal*	68.58% ± 0.41	0.227 ± 0.010	62.20% ± 0.82	0.322 ± 0.014
*ProtT5-beff*	52.26% ± 0.43	0.160 ± 0.016	71.47% ± 0.75	0.369 ± 0.016
*ConSeq-19equal*	**71.51% ± 0.39**	0.206 ± 0.010	50.70% ± 0.84	0.267 ± 0.012
*ConSeq blosum62*	54.32% ± 0.43	0.138 ± 0.016	63.81% ± 0.8	0.318 ± 0.014

^a^Data sets: The *PMD4k* data set contained 4 k proteins from the PMD (Kawabata et al. 1999); 74% of the SAVs were deemed effect in a binary classification. *DMS4* marks the first four human proteins (BRAC1, PTEN, TPMT, PPARG) for which we obtained comprehensive experimental DMS measurements along with a means of converting experimental scores into a binary version (effect/neutral) using synonyms. DMS4 results are shown for a threshold of 95%: the continuous effect scores were binarized by assigning the middle 95% of effect scores as neutral variants and SAVs resulting in effect scores outside this range as effect variants (Reeb et al. 2020). Methods: *SNAP2bin*: effect SNAP2 score > **−** 0.05, otherwise neutral; *VESPA*: effect score ≥ 0.5, otherwise neutral; *VESPAl*: effect score ≥ 0.5, otherwise neutral; *BLOSUM62*: negative BLOSUM62 scores predicted as effect, others as neutral; *ProtT5cons|ConSeq-19equal*: all 19 non-native SAVs predicted equally: effect if ProtT5cons|ConSeq predicted/labeled as conserved, otherwise neutral; *ProtT5beff|ConSeq-blosum62*: effect if ProtT5cons|ConSeq predicted/labeled as conserved and BLOSUM62 negative, otherwise neutral. ± values mark the 95% confidence interval (Eq. 12). For each column, if available, significantly best results are highlighted in bold

Most performances differed substantially between PMD4k and DMS4, i.e., the first four proteins (BRAC1, PTEN, TPMT, and PPARG) for which we had obtained large-scale experimental DMS measures that could be converted into a binary scale (effect/neutral). First, using BLOSUM62 to convert ProtT5cons into SAV-specific predictions outperformed the MSA-based conservation lookup from ConSeq, the expert method SNAP2 trained on PMD4k (Table 1: ProtT5beff highest rule-based), and the newly introduced VESPA. Second, combining the BLOSUM62 matrix with conservation also improved ConSeq (Table 1: ConSeq: *19equal* lower than *blosum62*). Third, ranking across different performance measures correlated much better than for PMD4k (Tables S1–S5). As the mapping from continuous DMS effect scores to binary labels might introduce systematic noise, we also investigated different thresholds for this mapping. However, results for DMS4 at intervals of 90% (Table S3) and 99% (Table S5) around the mean showed similar trends.

We trained a logistic regression (LR) ensemble (VESPA) on cross-validation splits replicated from the SNAP2 development set. For binary effect prediction, we introduced a threshold (≥ 0.5 effect, otherwise neutral) to the output scores of VESPA. When comparing VESPA and VESPAl (light version of VESPA) to the other methods on PMD4k, we observed a different picture than for the rule-based approaches. While SNAP2 still resulted in the highest MCC (0.28 ± 0.01), it was not significantly higher than that of VESPA and VESPAl (MCC: 0.274 ± 0.09 and 0.271 ± 0.09, respectively), and its development set overlapped with PMD4k. When evaluating the methods on DMS4, the best-performing method, VESPAl (MCC 0.405 ± 0.016), outperformed SNAP2 (MCC 0.204 ± 0.012) and VESPA (MCC 0.346 ± 0.014) as well as all rule-based methods (Table 1). We observed the same trends for other intervals (Tables S3–S5).

**Fig. 5:**
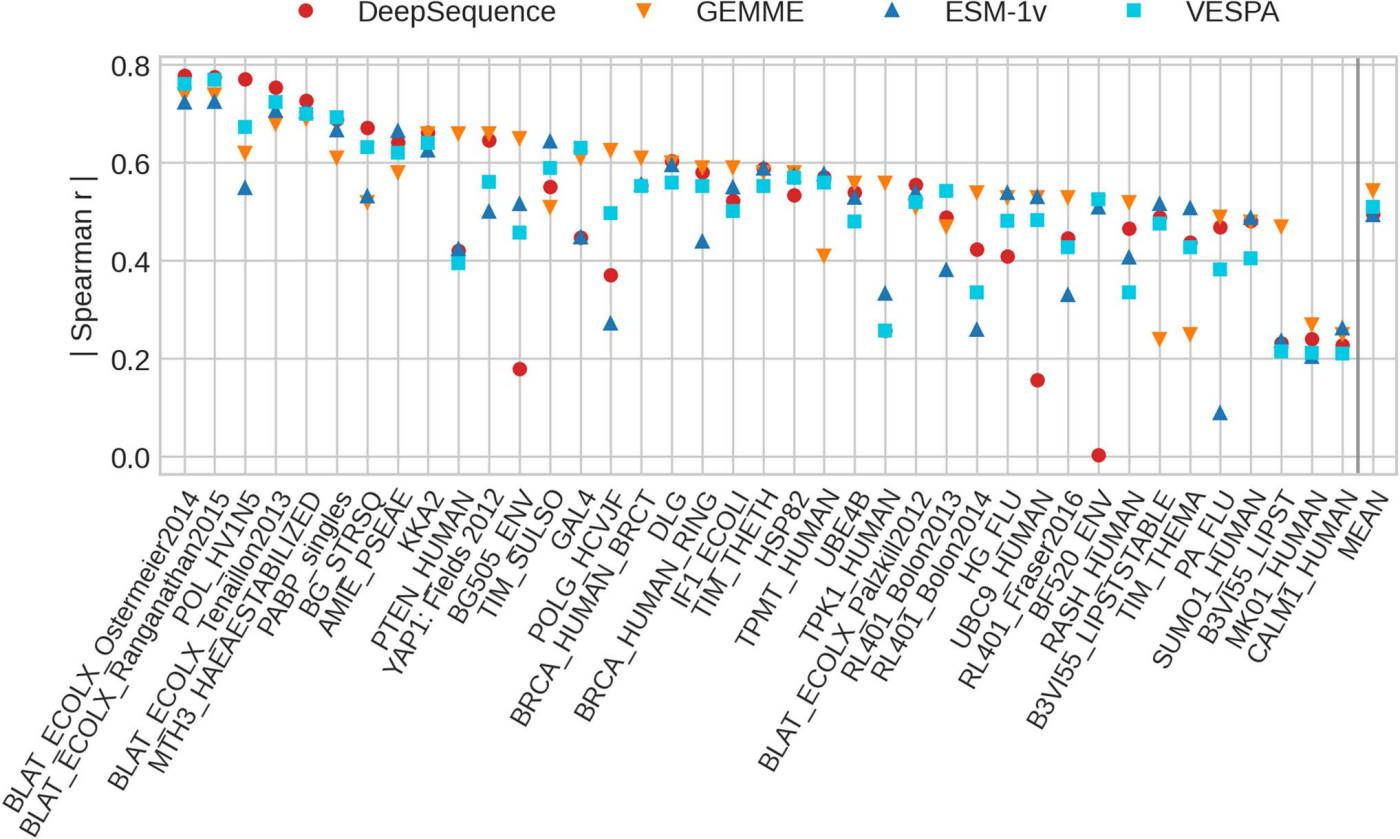
No SAV effect prediction consistently best on DMS data. Data: *DMS39* (39 DMS experiments gathered for the development of DeepSequence (Riesselman et al. 2018)); experiments sorted by the maximum absolute Spearman coefficient for each experiment. Methods: **a** *DeepSequence* trained an unsupervised model for each DMS experiment using only MSA input, i.e., no effect score labels were used (Riesselman et al. 2018); **b** *GEMME* inferred evolutionary trees and conserved sites from MSAs to predict effects (Laine et al. 2019); **c** *ESM-1v* correlated log-odds of substitution probabilities (Methods) with SAV effect magnitudes (Meier et al. 2021); **d** *VESPA* (this work) trained a logistic regression ensemble on binary SAV classification (effect/neutral) using predicted conservation (ProtT5cons), BLOSUM62 (Henikoff and Henikoff 1992), and log-odds of substitution probabilities from ProtT5 (Elnaggar et al. 2021) as input (without any optimization on DMS data). The values for the absolute Spearman correlation (Eq. 11) are shown for each method and experiment. The rightmost column shows the mean absolute Spearman correlation for each method. Although some experiments correlated much better (toward left) with predictions than others (toward right), the spread between prediction methods appeared high for both extremes; DeepSequence was the only method reaching a correlation of 0 for one experiment; another one and three experiments were predicted with correlations below 0.2 for ESM-1v and DeepSequence, respectively, while the vast number of the 4 × 39 predictions reached correlations above 0.4

**pLMs predicted SAV effect scores without MSAs.** Could VESPA, trained on binary effect data (Eff10k) capture continuous SAV effect scores measured by DMS? For ease of comparison with other methods, we chose all 39 DMS experiments (DMS39) with single SAV effect data assembled for the development of DeepSequence (Riesselman et al. 2018). Several methods have recently been optimized on DMS data, e.g., the apparent state-of-art (SOTA), DeepSequence trained on the MSAs of each of those 39 experiments. Another recent method using evolutionary information in a more advanced way than standard profiles from MSAs appears to reach a similar top level without machine learning, namely GEMME (Laine et al. 2019), and so does a method based on probabilities from pLMs, namely ESM-1v, without using MSAs. Comparing all those to VESPA, we could not observe a single method outperforming all others on all DMS39 experiments (Fig. 5). The four methods compared (two using MSAs: DeepSequence and GEMME, two using probabilities from pLMs instead of MSAs: ESM-1v and VESPA) reached Spearman rank correlations above 0.4 for 36 DMS experiments. In fact, for the 11 highest correlating out of the 39 experiments, predictions were as accurate as typically the agreement between two different experimental studies of the same protein (Spearman 0.65 (Reeb et al. 2020)).

**Fig. 6:**
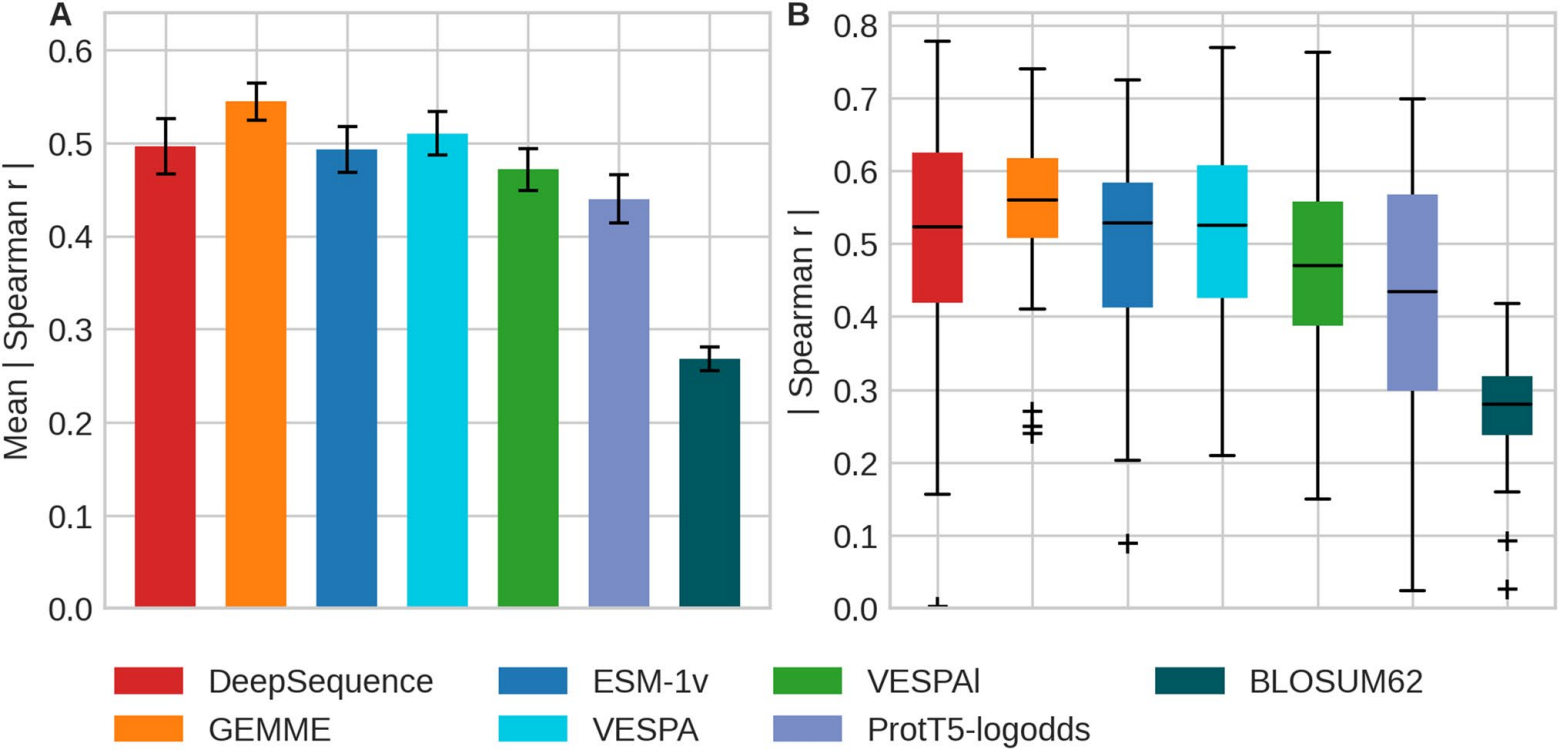
Spearman correlation between prediction and DMS experiment varied. Data and methods as for Fig. 5 with addition of: *VESPAl*: fast version of VESPA with input limited to ProtT5cons and BLOSUM62; *ProtT5-logodds*: raw log-odds from ProtT5 embeddings (Elnaggar et al. 2021); and raw *BLOSUM62* substitution scores (Henikoff and Henikoff 1992). Panel A: mean absolute Spearman correlation coefficient (Eq. 11) for each method over all 39 DMS experiments; error bars highlight 0.95 confidence interval (1.96 standard errors). Ignoring statistical significance, the numerical ranking would be: GEMME, VESPA, DeepSequence, ESM-1v, VESPAl, ProtT5-logodds, and BLOSUM62. However, the first four did not differ by any statistical significance, and while those ranked 5 and 6 differed from the best four, 5 was close to 4, and 6 close to 5; only BLOSUM62, the raw substitution scores compiled as background were clearly worst. Panel B: boxplots on absolute Spearman correlation coefficients (Eq. 11) for each method over the 39 DMS experiments. The medians are depicted as black horizontal bars; whiskers are drawn at the 1.5 interquartile range

GEMME had a slightly higher mean and median Spearman correlation (Eq. 11) than DeepSequence, ESM-1v, VESPA, and all others tested (Fig. 6A, Table 2). When considering the symmetric 95% confidence intervals (Eq. 12), almost all those differences were statistically insignificant (Fig. 6B) except for only using BLOSUM62. In terms of mean Spearman correlation, VESPA was slightly higher than DeepSequence, which was slightly higher than ESM-1v (Fig. 6A), but again neither was significantly better. The median Spearman correlation was equal for ESM-1v and VESPA and insignificantly lower for DeepSequence. The fastest method, VESPAl, reached lower Spearman correlations than all other major methods (Fig. 6). Ranking and relative performance after correcting for statistical significance were identical for Spearman and Pearson correlation (Table S6).

**Table 2:** Spearman correlation between SAV effect prediction and DMS experiments^a^

Method	Mean absolute ${{\varvec{r}}}_{{\varvec{S}}}$ (Eq. 11)	Median absolute ${{\varvec{r}}}_{{\varvec{S}}}$ (Eq. 11)
MSA-based		
*DeepSequence*	0.50 ± 0.03	0.52 ± 0.03
*GEMME*	0.53 ± 0.02	0.56 ± 0.02
*pLM-based*		
*ESM-1v*	0.49 ± 0.02	0.53 ± 0.02
*VESPA*	0.51 ± 0.02	0.53 ± 0.02
*VESPAl*	0.47 ± 0.02	0.47 ± 0.02

^a^Data sets: *DMS39* [39 DMS experiments gathered for the development of DeepSequence (Riesselman et al. 2018)] with 135,665 SAV scores. Methods: *DeepSequence*: AI trained on MSA for each of the DMS experiments (Riesselman et al. 2018); *GEMME*: using evolutionary information calculated from MSAs with few parameters optimized on DMS (Laine et al. 2019); *ESM-1v*: embedding-based prediction methods (Meier et al. 2021); *VESPA*: method developed here using logistic regression to combine predicted conservation (ProtT5cons), BLOSUM62 (Henikoff and Henikoff 1992) substitution scores, and log-odds from ProtT5 (Elnaggar et al. 2021); *VESPAl*: “light” version of VESPA using only predicted conservation and BLOSUM62 as input. ± values mark the standard error

For comparison, we also introduced two advances on a random baseline, namely the raw BLOSUM62 scores and the raw ProtT5 log-odds scores (Fig. 6; Fig. S7). BLOSUM62 was consistently and statistically significantly outperformed by all methods, while the ProtT5 log-odds averages were consistently lower, albeit not with statistical significance. As pLM-based methods were independent of MSAs, they predicted SAV scores for all residues contained in the DMS39 data sets, while, e.g., DeepSequence and GEMME could predict only for the subset of the residues covered by large enough MSAs. This was reflected by decreased coverage of methods relying on MSAs (DeepSequence and GEMME; Table S8). The Spearman correlation of ESM-1v, VESPA, and VESPAl for the SAVs in regions without MSAs was significantly lower than that in regions with MSAs available (Table S7).

**SAV effect predictions blazingly fast:** One important advantage of predicting SAV effects without using MSAs is the computational efficiency. For instance, to predict the mutational effects for all 19 non-native SAVs in the entire human proteome (all residues in all human proteins) took 40 min on one Nvidia Quadro RTX 8000 using VESPAl. In turn, this was 40 min more than using BLOSUM62 alone (nearly instantaneous), but this instantaneous BLOSUM62-based prediction was also much worse (Q2 for binary BLOSUM62 prediction worse than random, Table 1). In contrast, running methods such as SNAP2 (or ConSeq) required first to generate MSAs. Even the blazingly fast MMseqs2 (Steinegger and Söding 2017) needed about 90 min using batch-processing on an Intel Skylake Gold 6248 processor with 40 threads, SSD and 377 GB main memory. While VESPAl computed prediction scores within minutes for an entire proteome, VESPA and ESM-1v require minutes for some single proteins depending on sequence length, e.g., ESM-1v took on average 170 s per protein for the DMS39 set, while ProtT5 required on average 780 s. This originated from the number of forward passes required to derive predictions: while VESPAl needed only a single forward pass through the pLM to derive embeddings for conservation prediction, VESPA and ESM-1v (when deriving “masked-marginals” as recommended by the authors) required L forward passes with L being the protein length, because they corrupt one amino acid at a time and try to reconstruct it. The large difference in runtime between ESM-1v and ProtT5 originated from the fact that ESM-1v cropped sequences after 1022, reducing the strong impact of outliers, i.e., runtime of transformer-based models scales quadratically with sequence length, so while the shortest protein (71 residues) in the DMS39 set took only 5 s to compute, the longest (3033 residues) took 4.5 h to compute. We leave investigating the effect of splitting very long proteins into (overlapping) chunks to future work.

## Discussion

**Conservation predicted by embeddings without MSAs**. Even a simple logistic regression (LR) sufficed to predict per-residue conservation values from raw embeddings without using MSAs (Fig. 3, Table S1). Relatively shallow CNNs (with almost 100-times fewer free parameters than samples despite early stopping) improved over the LR to levels in predicting conservation only slightly below conservation assigned by ConSeq which explicitly uses MSAs (Fig. 3, Table S1). Did this imply that the pLMs extracted evolutionary information from unlabeled sequence databases (BFD (Steinegger and Söding 2018) and UniProt (The UniProt Consortium 2021))? The answer might be more elusive than it seems. The methodology (pLMs) applied to predict conservation never encountered any explicit information about protein families through MSAs, i.e., the pLMs used here never had an explicit opportunity to pick up evolutionary constraints from related proteins. The correlation between substitution probabilities derived from pLMs and conservation (Fig. 2) might suggest that pLMs implicitly learned evolutionary information.

A possible counterargument builds around the likelihood to pick up evolutionary constraints. The pLM clearly learned the reconstruction of more frequent amino acids much better than that of less frequent ones (Fig. S5). Unsurprisingly, AI is pushed most in the direction of most data. In fact, the differences between amino acid compositions were relatively small (less than factor of 10), suggesting that even an event occurring at one-tenth of the time may challenge pLMs. If the same pLM has to learn the evolutionary relation between two proteins belonging to the same family, it has to effectively master an event happening once in a million (assuming an average family size of about 2.5 k—thousand—in a database with 2.5b—billion—sequences). How can the model trip over a factor of 10^1^ and at the same time master a factor of 10^6^? Indeed, it seems almost impossible. If so, the pLM may not have learned evolutionary constraints, but the type of *bio-physical* constraint that also constrain evolution. In this interpretation, the pLM did not learn evolution, but the constraints “written into protein sequences” that determine which residue positions are more constrained.

In fact, one pLM used here, namely ProtT5, has recently been shown to explicitly capture aspects of long-range inter-residue distances directly during pre-training, i.e., without ever being trained on any labeled data pLMs pick up structural constraints that allow protein 3D structure prediction from single protein sequences (Weißenow et al. 2021). Another explanation for how ProtT5 embeddings capture conservation might be that pLMs picked up signals from short, frequently re-occurring sequence/structure motifs such as localization signals or catalytic sites that are more conserved than other parts of the sequence. If so, the pLM would not have to learn relationship between proteins but only between fragments, thereof reducing the factor 10^6^ substantially. We could conceive of these motifs resembling some evolutionary nuclei, i.e., fragments shorter than structural domains that drove evolution (Alva et al. 2015; Ben-Tal and Lupas 2021; Kolodny 2021). Clearly, more work will have to shed light on the efficiency of (p)LMs in general (Bommasani et al. 2021).

**Transformer-based pLMs best?** We have tested a limited set of pLMs, largely chosen, because those had appeared to perform better than many other methods for a variety of different prediction tasks. Does the fact that in our hands Transformer-based pLMs worked best to predict residue conservation and SAVs imply that those will generally outperform other model types? By no means. While we expect that the about twenty approaches that we have compared in several of our recent methods (including the following 13: ESM-1[b|v] Meier et al. 2021; Rives et al. 2021), ProSE[*|DLM|MT] (Bepler and Berger 2019b, 2021), Prot[Albert|Bert|Electra|Vec|T5|T5XL|T5XLNet|T5XXL] (Elnaggar et al. 2021; Heinzinger et al. 2019) provided a somehow representative sampling of the existing options, our conclusions were only valid for embeddings extracted in a generic way from generic pLMs without any bearing on the methods underlying those pLMs.

**Predicted conservation informative about SAV effects:** DMS data sets with comprehensive experimental probing of the mutability landscape (Hecht et al. 2013) as, e.g., collected by MaveDB (Esposito et al. 2019) continue to pose problems for analysis, possibly due to a diversity of assays and protocols (Livesey and Marsh 2020; Reeb et al. 2020). Nevertheless, many such data sets capture important aspects about the susceptibility to change, i.e., the mutability landscape (Hecht et al. 2013). As always, the more carefully selected data sets become, the more they are used for the development of methods and therefore no longer can serve as independent data for assessments (Grimm et al. 2015; Reeb et al. 2016). Avoiding the traps of circularity and over-fitting by skipping training, our non-parametric rule-based approaches (ProtT5cons and ProtT5beff) suggested that predictions of SAV effects (by simply assigning “effect” to those SAVs where ProtT5cons predicted conserved and the corresponding BLOSUM62 value was negative) outperformed ConSeq with MSAs using the same idea, and even the expert effect prediction method SNAP2 (Fig. 4, Table 1).

Strictly speaking, it might be argued that one single free parameter was optimized using the data set, because for the PMD4k data set, the version that predicted the same effect for all 19-SAVs appeared to outperform the SAV-specific prediction using BLOSUM62 (*19equal* vs *blosum62* in Fig. 4 and Table 1). However, not even the values computed for PMD4k could distract from the simple fact that not all SAVs are equal, i.e., that regardless of model performance, *19equal* will not be used exclusively for any method. In fact, the concept of combining predictions with BLOSUM62 values has been shown to succeed for function prediction before (Bromberg and Rost 2008; Schelling et al. 2018) in that sense it was arguably not an optimizable hyperparameter. Embeddings predicted conservation (Fig. 3); conservation predicted SAV effects (Fig. 4). Did this imply that embeddings captured evolutionary information? Once again, we could not answer this question either way directly. To repeat: our procedure/method never used information from MSAs in any way. Could it have implicitly learned this? To repeat the previous speculation: embeddings *might* capture a reality that constrains what can be observed in evolution, and this reality is exactly what is used for the part of the SAV effect prediction that succeeds. If so, we would argue that our simplified method did not succeed, because it predicted conservation without using MSAs, but that it captured positions biophysically “marked by constraints”, i.e., residues with higher contact density in protein 3D structures (Weißenow et al. 2021). This assumption would explain how predicted conservation (ProtT5cons) not using evolutionary information could predict SAV effects better than a slightly more correct approach (ConSeq) using MSAs to extract evolutionary information (Fig. 4: ProtT5cons vs. ConSeq).

**Substitution probabilities from pLMs capture aspects measured by DMS experiments:** Using embeddings to predict SAV effects through conservation prediction succeeded but appeared like a detour. ESM-1v (Meier et al. 2021) pioneered a direct path from reconstruction/substitution probabilities of pLMs to SAV effect predictions. When comparing the ESM-1v encoder-based with the ProtT5 encoder–decoder-based Transformer, we encountered surprising results. Previously, ProtT5 usually performed at least on par with previous versions of ESM (e.g., ESM-1b (Rives et al. 2021)) or outperformed them (Elnaggar et al. 2021). In contrast, the substitution probabilities of ProtT5 were clearly inferior to those from ESM-1v in their correlation with the 39 DMS experiments (Fig. 6). This reversed trend might have resulted from a combination of the following facts: (1) ProtT5 is a single model, while ESM-1v is an ensemble of five pLMs potentially leading to a smoother substitution score. (2) ESM-1v was trained on UniRef90 instead of BFD/UniRef50 (ProtT5) possibly providing a broader view on the mutability landscape of proteins. In fact, the ESM-1v authors showed a significant improvement when pre-training on UniRef90 instead of UniRef50 (Rives et al. 2021). (3) ESM-1v is a BERT-style, encoder-based Transformer, while ProtT5 is based on T5’s encoder-decoder structure. In previous experiments (Elnaggar et al. 2021), we only extracted embeddings from ProtT5’s encoder (e.g., ProtT5cons is based on encoder embeddings), because its decoder fell significantly short in all experiments. However, only T5’s decoder can output probabilities, so we had to fall back to ProtT5’s decoder for SAV effect predictions. This discrepancy of encoder and decoder performance can only be sketched here. In short, encoder-based transformer models always *see* the context of the whole sequence (as does ProtT5 ‘s encoder and ESM-1v), while decoder-based transformer models (such as ProtT5’s decoder or GPT (Radford et al. 2019)) *see* only single-sided context, because they are generating text (sequence-to-sequence models (Sutskever et al. 2014)). This is crucial for translation tasks, but appeared sub-optimal in our setting. Despite this shortcoming in performance, we trained VESPA based on log-odds derived from ProtT5 substitution probabilities, mainly because we started this work before the release of ESM-1v. Also, we hoped for synergy effects when implementing VESPA into the PredictProtein webserver, because ProtT5 is already used by many of our predictors. Finding the best combination of pLM substitution probabilities for SAV effect prediction will remain subject for future work.

**Fast predictions save computing resources?** Our simple protocol introduced here enabled extremely efficient, speedy predictions. While pre-training pLMs consumed immense resources (Elnaggar et al. 2021), this was done in the past. The new development here was the models for the 2nd level supervised transfer learning. Inputting ProtT5 embeddings to predict residue conservation (ProtT5cons) or SAV effects (VESPA/VESPAl) for predictions in the future will consume very little additional resources. When running prediction servers such as PredictProtein (Bernhofer et al. 2021) queried over 3000 times every month, such investments could be recovered rapidly at seemingly small prices to pay even if performance was slightly reduced. How to quantify this? At what gain in computing efficiency is which performance reduction acceptable? Clearly, there will not be one answer for all purposes, but the recent reports on climate change strongly suggest to begin considering such questions.

**Quantitative metrics for hypothetical improvements over MSA-based methods?** If methods using single sequences without MSAs perform as well as, or even better than, SOTA methods using MSAs, could we quantify metrics measuring the hypothetical improvements from embeddings? This question raised by an anonymous reviewer opens an interesting new perspective. Gain in speed, reduction of computational costs clearly could evolve as one such metric. A related issue is related to protein design: for some applications, the difference in speed might open new doors. Although we have no data to show for others, we could imagine yet another set of metrics measuring the degree to which embedding-based methods realize more protein-specific than family averaged predictions.

## Conclusions

Embeddings extracted from protein Language Models (pLMs, Fig. 1), namely from ProtBert and ProtT5 (Elnaggar et al. 2021) and ESM-1b (Rives et al. 2021), contain information that sufficed to predict residue conservation in protein families without using multiple sequence alignments (MSAs, Fig. 3). Such predictions of conservation combined with BLOSUM62 scores predicted the effects of sequence variation (single amino acid variants, or SAVs) without optimizing any additional free parameter (*ProtT5beff*, Fig. 6). Through further training on binary experimental data (effect/neutral), we developed *VESPA*, a relatively simple, yet apparently successful new method for SAV effect prediction (Fig. 4). This method even worked so well on non-binary data from 39 DMS experiments that without ever using such data nor ever using MSAs; VESPA appeared competitive with the SOTA (Fig. 5, Fig. 6), although for SAV effect predictions, embedding-based methods are still not yet outperforming the MSA-based SOTA as for other prediction tasks (Elnaggar et al. 2021; Littmann et al. 2021a, b, c; Stärk et al. 2021). Embedding-based predictions are blazingly fast, thereby they save computing, and ultimately energy resources when applied to daily sequence analysis. In combination, our results suggested that the major signal captured by variant effect predictions originates from some bio-physical constraint revealed by raw protein sequences. The ConSurf10k dataset is available at https://doi.org/10.5281/zenodo.5238537. For high-throughput predictions, methods are available through bio_embeddings (Dallago et al. 2021). For single queries VESPA and ProtT5cons will be made available through the PredictProtein server (Bernhofer et al. 2021). VESPA and VESPAl are also available from github at https://github.com/Rostlab/VESPA.

## Supplementary Information

Below is the link to the electronic supplementary material.

Supplementary file1 (PDF 891 KB)

Supplementary file2 (XLSX 31 KB)

Supplementary file3 (XLSX 16446 KB)

## Abbreviations

AI: Artificial intelligence
AUC: Area under the curve
BFD: Big Fantastic Database
CNN: Convolutional neural network
DL: Deep learning
EI: Evolutionary information
DMS: Deep mutational scanning
FNN: Feed forward neural network
GoF: Gain-of-function SAV
LoF: Loss-of-function SAV
LM: Language model
LR: Logistic regression
MAVE: Multiplexed Assays of Variant Effect
MCC: Matthews correlation coefficient
ML: Machine learning
MSA: Multiple sequence alignments
NLP: Natural language processing
OMIM: Online Mendelian Inheritance in Man
PDB: Protein Data Bank
pLM: Protein Language Model (used here: ESM-1b/1v: ProtBERT: ProtT5)
PMD: Protein mutant database
ProtT5beff: Rule-based method developed here using ProtT5 embeddings to predict binary SAV effects from single sequences
ProtT5cons: Method developed here using ProtT5 embeddings to predict residue conservation from single sequences optimizing a CNN on the unchanged pre-trained ProtT5
ReLU: Rectified linear unit
ROC: Receiver-operating characteristic
SAV: Single amino acid variant (also known as SAAV or nsSNP: or missense mutation/variant)
SOTA: State-of-the-art
SSD: Solid State Drive
SVM: Support Vector Machine
VESPA: Method developed here for Variant Effect Score Prediction without Alignments
VESPAl: Light VESPA: less accurate but faster
