# Supplementary material for: Embeddings from protein language models predict conservation and variant effects
Source: Hum Genet. 2021 Dec 30;141(10):1629–47. doi: 10.1007/s00439-021-02411-y (PMC8716573; doi:10.1007/s00439-021-02411-y)
Supplement: Supplementary file 1 — Supplementary file1 (PDF 891 KB) [file 439_2021_2411_MOESM1_ESM.pdf]

Supporting online material for: Embeddings from protein language models predict conservation and variant effects

Céline Marquet[1,2*†], Michael Heinzinger[1,2†], Tobias Olenyi[1,2], Christian Dallago[1,2], Kyra Erckert[1,2], Michael Bernhofer[1,2], Dmitrii Nechaev[1,2], & Burkhard Rost[1,3]

1 TUM (Technical University of Munich) Department of Informatics, Bioinformatics & Computational Biology - i12, Boltzmannstr. 3, 85748 Garching/Munich, Germany
2 TUM Graduate School, Center of Doctoral Studies in Informatics and its Applications (CeDoSIA), Boltzmannstr. 11, 85748 Garching, Germany
3 Institute for Advanced Study (TUM-IAS), Lichtenbergstr. 2a, 85748 Garching/Munich, Germany & TUM School of Life Sciences Weihenstephan (TUM-WZW), Alte Akademie 8, Freising, Germany
* Corresponding author: celine.marquet@tum.de, http://www.rostlab.org/
† Céline Marquet and Michael Heinzinger contributed equally to this work
Tel: +49-289-17-811 (email rost: assistant@rostlab.org)

Table of Contents for Supporting Online Material

SHORT DESCRIPTION OF SUPPORTING ONLINE MATERIAL................................ 2
MATERIAL.. 3
Fig. S1: F1 and MCC scores for different thresholds when binarizing 9-class conservation predictions on ConSurf 3
Fig. S2: Confusion matrix for 2-class conservation prediction on ConSurf 4
Fig. S3: Confusion matrix for 9-class conservation prediction on ConSurf 5
Fig. S4: Confusion matrix for amino acid reconstruction on ConSurf 6
Fig. S5: Frequent amino acids more likely to be reconstructed by ProtBert than rare amino acids 7
Fig. S6: Distribution of ProtBert's reconstruction probabilities 8
Fig. S7: Absolute Spearman correlation coefficient of VESPA, VESPAl, ProtT5-logodds and BLOSUM62 on DMS39.. 9
Table S1: Performance comparison for predicting residue conservation on ConSurf10k* 10
Table S2: Performance comparison for predicting binary SAV effects on PMD* 11
Table S3: Performance comparison for predicting SAV effects on DMS4 (90%)*.................................. 12
Table S4: Performance comparison for predicting SAV effects on DMS4 (95%)*.................................. 13
Table S5: Performance comparison for predicting SAV effects on DMS4 (99%)*.................................. 14
Table S6: Pearson correlation between SAV effect prediction and DMS39 experiments*...................... 15
Table S7: pLM-based SAV effect prediction: comparison of Spearman correlation for DMS39 scores with and without available MSAs* 16
Table S8: Total number of SAV effect predictions and coverage of DMS scores on DMS39*................. 17
REFERENCES FOR SUPPORTING ONLINE MATERIAL 18

Short description of Supporting Online Material

In this Supporting Online Material (SOM), we provide an extended analysis of our 9- (Fig. S3, Table S1) and 2-class (Fig. S2, Table S1) residue conservation prediction performance and further substantiate the choice of our threshold for binary conservation prediction (Fig. S1). Furthermore, we evaluate ProtBert's (Elnaggar et al. 2021) amino acid reconstruction performance to facilitate our understanding of the model's mistakes during token reconstruction (Fig. S4, Fig. S5). The distribution of those probabilities is also added (Fig. S6). Additionally, we provide a more detailed overview of binary SAV effect prediction performance on data from PMD4k (Kawabata et al. 1999) (Table S2) and from four DMS experiments (DMS4) of human proteins (Findlay et al. 2018; Majithia et al. 2016; Matreyek et al. 2018) when binarized for different thresholds (Table S3, Table S4, Table S5). Finally, we complement our binary SAV effect analysis by correlating (Pearson's R) continuous SAV effect score magnitudes from 39 in-vitro DMS experiments (DMS39) with various effect score predictors (DeepSequence, GEMME, VESPA, VESPAl).

A detailed overview of the experiments within DMS39 as well as method evaluations are available in the excel file DMS39_datasets_correlation.xlsx of the Supporting Supplementary Information (SI). Furthermore, predictions per experiment are available in DMS39_predictions.xlsx.

Material

Fig. S1: F1 and MCC scores for different thresholds when binarizing 9-class conservation predictions on ConSurf

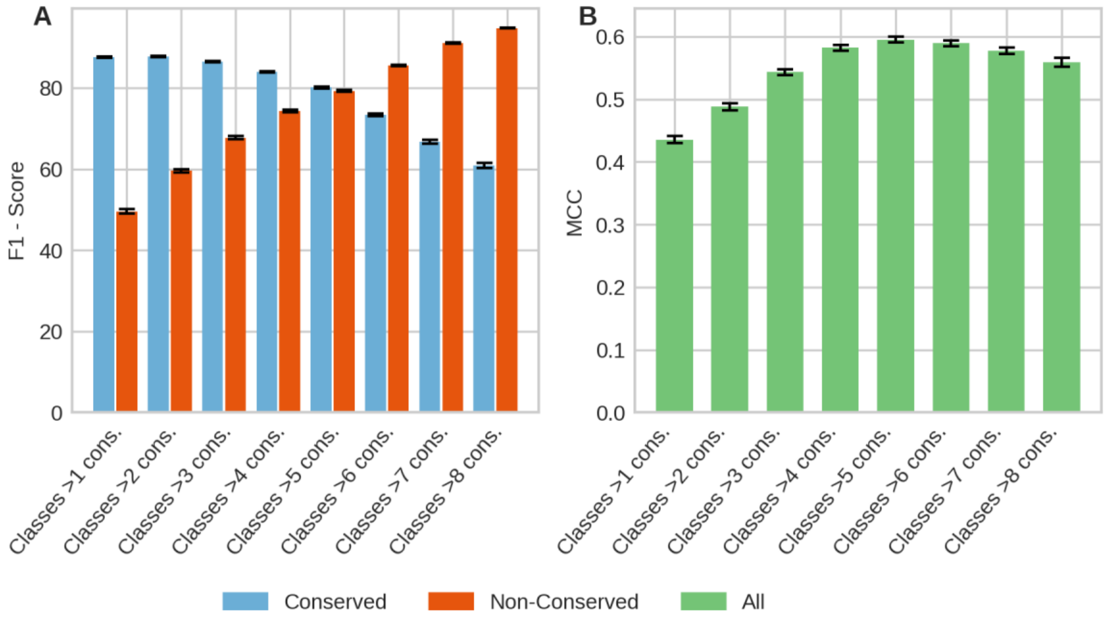

Evaluation on the Consurf10k cross-validation set, which consists of 555 proteins annotated with ConSurf (Ben Chorin et al. 2020) 9-class conservation scores. Embeddings from ProtT5 (Elnaggar et al. 2021) were used as input to a convolutional neural network (CNN; method dubbed ProtT5cons) to predict 9-class conservation scores. For binarizing the 9 classes into "conserved" and "non-conserved" different thresholds were used. The thresholds range from "Classes >1 cons." with non-conserved being "1" and conserved being "2-9" up to "Classes >8 cons." with non-conserved being "1-8" and conserved being "9". Panel A shows $F1_+$ (Eqn. 6) for "conserved" predictions of ProtT5cons in blue and $F1_-$ (Eqn. 7) for "non-conserved" predictions in red at different thresholds. Panel B shows MCC (Eqn. 8) scores at different thresholds. The threshold of "5" (non-conserved "1-5" and conserved "6-9") had the highest MCC value and the best F1 scores when maximizing for both positive and negative classes. For detailed dataset and method descriptions see Datasets and Methods. Black bars mark the 95% confidence interval (Eqn. 12).

Fig. S2: Confusion matrix for 2-class conservation prediction on ConSurf

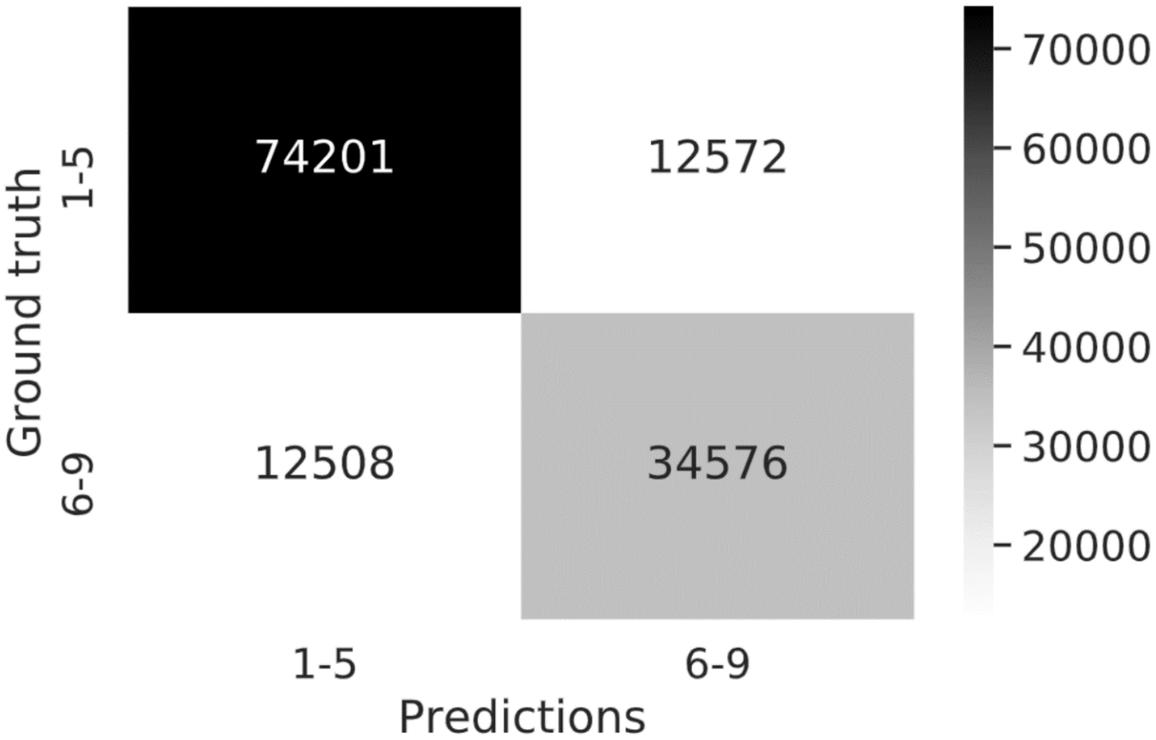

Evaluation of residue conservation prediction on the final ConSurf10k hold-out test set (519 protein sequences, 49.2% conserved, 50.8% non-conserved residues). 9-class predictions were binarized by mapping the classes to non-conserved (1-5) and conserved (6-9). Embeddings from ProtT5 were used as input to a convolutional neural network (CNN; method dubbed ProtT5cons). For detailed dataset and method descriptions see Datasets and Methods.

Fig. S3: Confusion matrix for 9-class conservation prediction on ConSurf

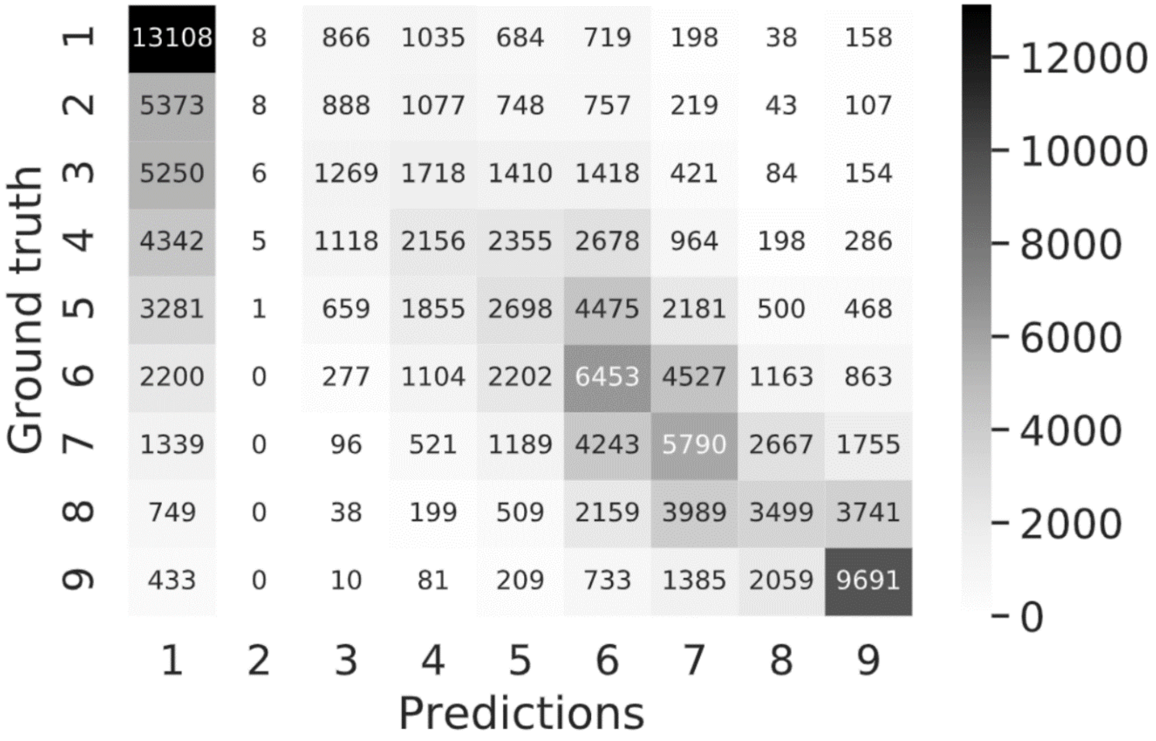

Evaluation of 9-class residue conservation prediction on the final ConSurf10k hold-out test set (519 protein sequences) with 1 being highly non-conserved and 9 being highly conserved. Embeddings from ProtT5 were used as input to a convolutional neural network (CNN; method dubbed ProtT5cons). Most wrong predictions fall into neighboring conservation bins highlighted by the shade along the diagonal. The network did not learn to distinguish between the most variable classes 1 and 2. Instead, the network learnt to predict the majority class (class 1). For detailed dataset and method descriptions see Datasets and Methods.

Fig. S4: Confusion matrix for amino acid reconstruction on ConSurf

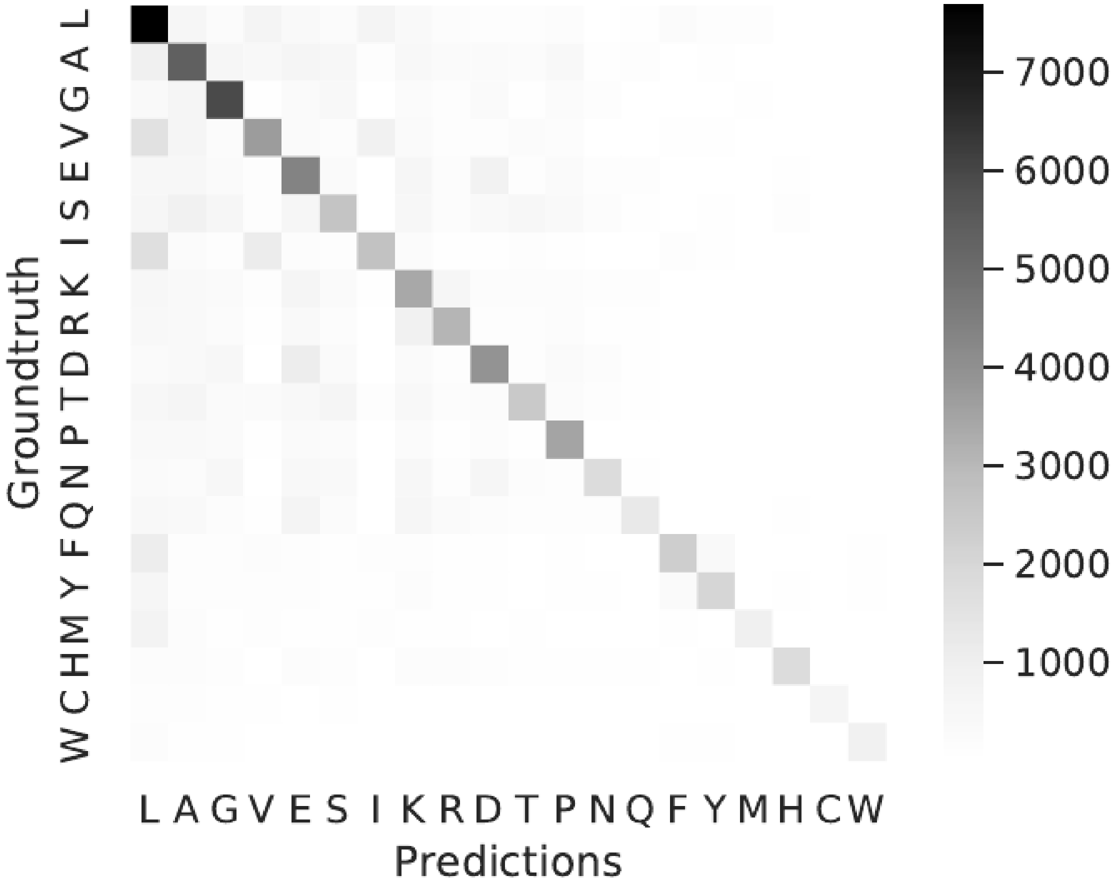

Evaluation of ProtBert's amino acid reconstruction performance on the final ConSurf10k hold-out test set (519 protein sequences). During pre-training, ProtBert was optimized on reconstructing corrupted input tokens from non-corrupted sequence context (masked language modeling). Here, we corrupted and reconstructed all residues in all proteins of the ConSurf10 hold-out test set, one residue at a time. This confusion matrix shows the performance of ProtBert's ability to reconstruct the correct amino acid when picking the amino acid reconstructed with the highest probability. In total, ProtBert was able to reconstruct 43.5% of the amino acids in this set correctly, compared to a random baseline of 9.4% when picking always the most frequent amino acid.

Fig. S5: Frequent amino acids more likely to be reconstructed by ProtBert than rare amino acids

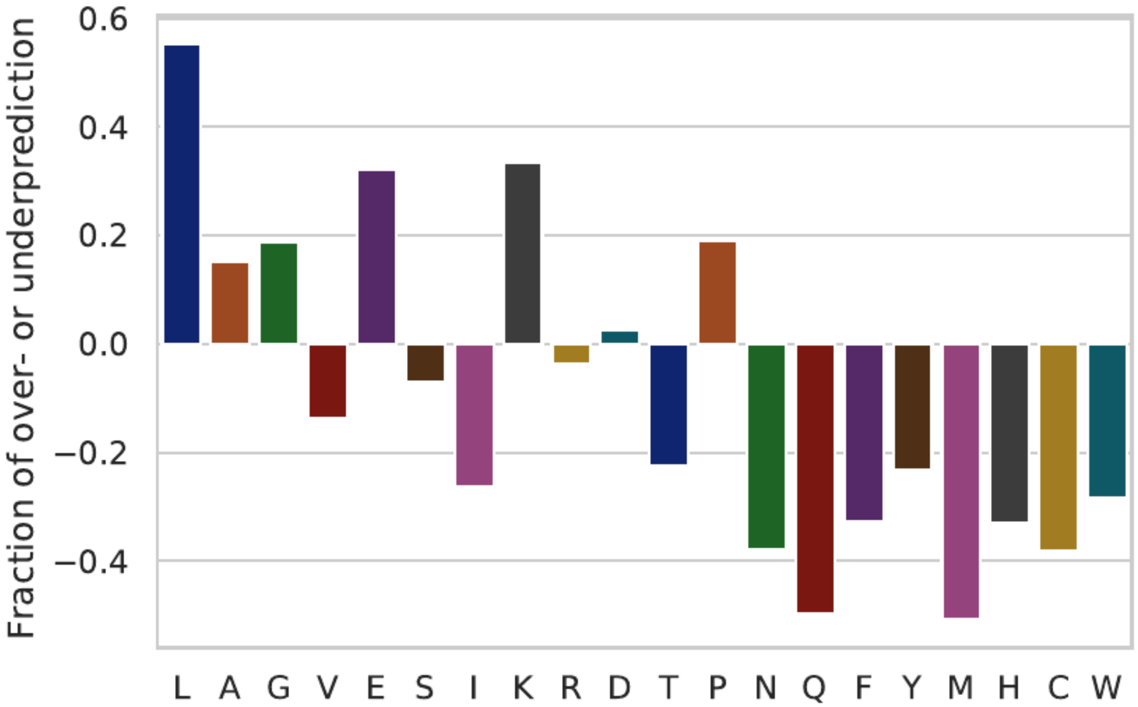

Here, we illustrated the over- and under-prediction of different amino acid types from ProtBert's mask reconstruction probabilities (Elnaggar et al. 2021). In brief, we used the ConSurf10k hold-out set (519 protein sequences) to corrupted one residue at a time and used ProtBert to reconstruct it from non-corrupted sequence context. We divided the number of predictions per amino acid type (sum over rows in Fig. S4) to the number of true occurrences of this amino acid type in the hold out set (sum over columns in Fig. S4) and subtracted 1. As a result, values around 0 implied that an amino acid was predicted with the same frequency as it appeared in the test set, while values around 1 implied that observing the prediction of this amino acid was twice as likely as expected (compiled according to background frequency in dataset). Negative values, in turn, implied predicting this amino acid type less often than expected by chance. Amino acids shown on the x-axis are ordered by their background frequency in the data set (left: most frequent; right: least frequent).

Fig. S6: Distribution of ProtBert's reconstruction probabilities

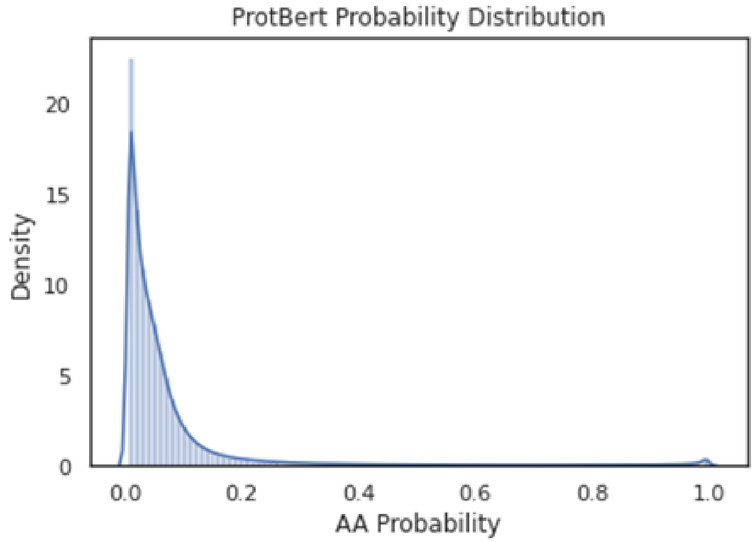

In its original publication, ProtBert was trained on masked language modeling, i.e., reconstructing corrupted input tokens from non-corrupted sequence context. Here, we corrupted and reconstructed, one residue at a time, all residues of the entire ConSurf10k dataset. As a result, ProtBert returns a probability for each of the 20 amino acids for each residue position. The distribution shows that ProtBert tends to assign very high probabilities to very few amino acids (small peak around 1) while assigning relatively low probabilities to the other 19 amino acids.

Fig. S7: Absolute Spearman correlation coefficient of VESPA, VESPAl, ProtT5-logodds and BLOSUM62 on DMS39

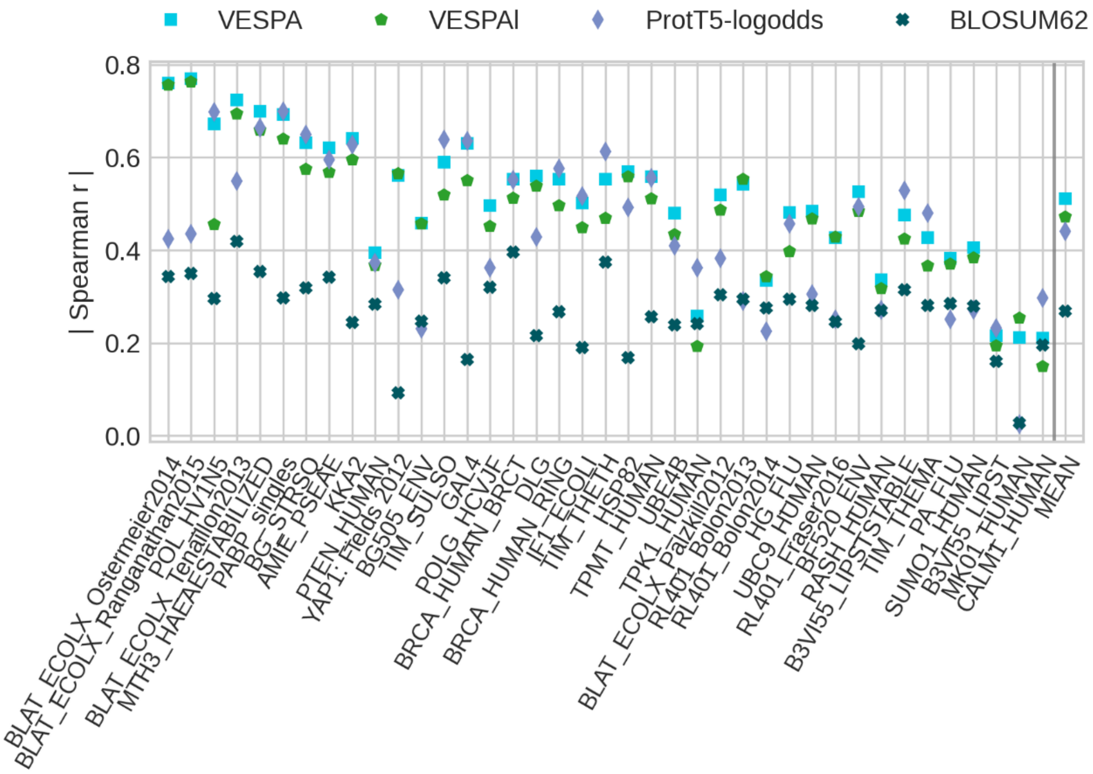

Data: DMS39 (39 DMS datasets, subset of experiments gathered for development of DeepSequence (Riesselman et al. 2018)), experiments are sorted by the maximum absolute Spearman coefficient (Eqn. 11) regardless of method; Methods: a) VESPA: Logistic Regression Ensemble for SAV effect prediction using residue conservation prediction from ProtT5cons, substitution scores from BLOSUM62, and the log-odds ratio of substitution probabilities from ProtT5 (dubbed ProtT5-logodds); b) VESPAl: fast version of VESPA using only residue conservation prediction from ProtT5cons, and substitution scores from BLOSUM62; c) ProtT5-logodds: log-odds ratio of the substitution probability for a wild-type amino acid a specific mutant at the same position (see Methods – Substitution probabilities for more details); d) BLOSUM62: context-independent substitution scores from BLOSUM62. Absolute Spearman correlation (Eqn. 11) is shown for each method and experiment. The rightmost column shows the mean absolute Spearman correlation for each method.

Table S1: Performance comparison for predicting residue conservation on ConSurf10k*

Input	Method	Q9 (Eqn. 9)	Q2 (Eqn. 1)	F1 (Eqn. 6)	MCC (Eqn. 8)	r_P (Eqn. 10)
Random	-	12.4% ± 0.2	49.9% ± 0.4	51.6% ± 0.4	0.000 ± 0.006	0.000 ± 0.002
ESM-1b embeddings	LR	30.8% ± 0.4	79.0% ± 0.1	69.1% ± 0.2	0.534 ± 0.003	0.652 ± 0.001
	FNN	32.4 ± 0.4	79.9 ± 0.1	71.4% ± 0.2	0.563 ± 0.003	0.677 ± 0.002
	CNN	32.4 ± 0.4	80.2 ± 0.1	71.9% ± 0.2	0.563 ± 0.003	0.676 ± 0.002
ProtBert embeddings	LR	29.6% ± 0.2	75.6% ± 0.4	76.2% ± 0.4	0.514 ± 0.006	0.627 ± 0.004
	FNN	30.5% ± 0.2	76.7% ± 0.4	77.3% ± 0.4	0.536 ± 0.006	0.642 ± 0.004
	CNN	31.2% ± 0.2	76.7% ± 0.4	77.8% ± 0.4	0.540 ± 0.004	0.654 ± 0.004
ProtT5 embeddings	LR	32.1% ± 0.2	78.6% ± 0.4	79.0% ± 0.4	0.573 ± 0.006	0.704 ± 0.001
	FNN	33.5% ± 0.2	79.4% ± 0.4	79.8% ± 0.4	0.590 ± 0.002	0.715 ± 0.002
	CNN	33.4% ± 0.2	79.7% ± 0.4	80.1% ± 0.4	0.596 ± 0.006	0.722 ± 0.004
MMseqs2, PSI-BLAST	ConSeq	33.8% ± 0.2	80.2% ± 0.4	80.9% ± 0.4	0.608 ± 0.006	0.751 ± 0.004

*Conservation prediction evaluation on the final ConSurf10k hold-out test set (519 protein sequences). 9-class predictions were binarized by mapping the classes to non-conserved (1-5) and conserved (6-9). Embeddings from three protein LMs (ESM-1b, ProtBert and ProtT5) were used as input to three classifiers (Logistic regression (LR), feed-forward neural network (FNN) and convolutional neural network (CNN)). Additionally, we compared to an existing solution that identifies residue conservation using evolutionary information from multiple-sequence alignments (ConSeq). We found that the CNN trained on ProtT5 embeddings reaches a performance within the confidence interval of ConSeq. r_P refers to the Pearson correlation coefficient. For detailed dataset and method descriptions see Datasets and Methods. ± values mark the 95% confidence interval (Eqn. 12).

Table S2: Performance comparison for predicting binary SAV effects on PMD*

Method	$F1_{effect}$ (Eqn. 6)	$F1_{neutral}$ (Eqn. 7)	Q2 (Eqn. 1)	MCC (Eqn. 8)
Random	73.80% ± 0.71	26.31% ± 1.41	61.08% ± 0.41	-0.002 ± 0.016
**Supervised Methods**				
SNAP2bin	79.53% ± 0.17	48.21% ± 0.37	70.66% ± 0.39	0.28 ± 0.010
VESPA	71.29% ± 0.41	50.00% ± 0.66	63.52% ± 0.43	0.274 ± 0.009
VESPAl	70.74% ± 0.42	49.87% ± 0.63	63.04% ± 0.43	0.271 ± 0.009
**Rule-Based Methods**				
BLOSUM62bin	66.85% ± 0.21	35.36% ± 0.33	56.17% ± 0.43	0.049 ± 0.010
ProtT5cons 19equal	78.11% ± 0.16	44.36% ± 0.36	68.58% ± 0.41	0.227 ± 0.010
ProtT5beff	58.07% ± 0.25	44.59% ± 0.3	52.26% ± 0.43	0.16 ± 0.016
ConSeq 19equal	**81.49% ± 0.15**	38.24% ± 0.4	**71.51% ± 0.39**	0.206 ± 0.010
ConSeq blosum62	61.94% ± 0.23	42.89% ± 0.3	54.32% ± 0.43	0.138 ± 0.016

*Binary SAV effect prediction evaluation on the PMD4k dataset consisting of 51,817 binary annotated variants (neutral: 13,638, effect: 38,179) in 4,061 proteins. ConSeq failed for 122 proteins and SNAP2 failed for 4 proteins. Methods developed here using either binarized conservation prediction alone (ProtT5cons 19equal) or BLOSUM62 scores in addition (ProtT5beff) are compared against different existing baselines: a) BLOSUM62bin relies on substitution scores alone, b) ConSeq 19equal relies on binarized conservation scores alone and c) ConSeq blosum62 combines the two aforementioned methods. Further, we evaluate the logistic regression ensemble for Variant Effect Score Prediction without Alignments (VESPA) introduced here and its computationally more efficient counterpart (VESPAl). Finally, we compare to one established SAV effect predictor (SNAP2bin). For detailed dataset and method descriptions see Datasets and Methods. ± values mark the 95% confidence interval (Eqn. 12). For each measure, significantly best results are highlighted in bold.

Table S3: Performance comparison for predicting SAV effects on DMS4 (90%)*

Method	$F1_{effect}$ (Eqn. 6)	$F1_{neutral}$ (Eqn. 7)	Q2 (Eqn. 1)	MCC (Eqn. 8)
Random	33.91% ± 1.23	66.35% ± 0.8	55.4% ± 0.89	0.003 ± 0.017
**Supervised Methods**				
SNAP2bin	54.97% ± 0.97	41.64% ± 1.13	49.16% ± 0.82	0.225 ± 0.013
VESPA	60.39% ± 1.03	69.90% ± 0.86	65.79% ± 0.81	0.352 ± 0.015
VESPAl	60.79% ± 1.14	77.29% ± 0.70	71.24% ± 0.76	**0.386 ± 0.016**
**Rule-Based Methods**				
BLOSUM62bin	52.64% ± 0.97	46.47% ± 1.03	49.74% ± 0.86	0.166 ± 0.015
ProtT5cons 19equal	58.89% ± 1.07	68.28% ± 0.85	64.19% ± 0.78	0.324 ± 0.016
ProtT5beff	58.65% ± 1.17	76.49% ± 0.71	70.02% ± 0.77	0.355 ± 0.016
ConSeq 19equal	56.99% ± 0.96	54.27% ± 1.03	55.67% ± 0.84	0.274 ± 0.014
ConSeq blosum62	57.89% ± 1.13	69.61% ± 0.84	64.69% ± 0.84	0.311 ± 0.016

*_DMS4_ dataset consisting of binary annotated variants in 4 human proteins (BRAC1, PTEN, TPMT, PPARG). Annotations were created by considering the middle 90% of effect scores as neutral SAVs (Reeb et al. 2020). All variants outside these thresholds were considered effect (neutral SAVs: 8,926, deleterious effect SAVs: 4,545). The results for other thresholds (95%, 99%) can be found in SOM Tables 4 and 5 (trends are similar). Methods developed here using either binarized conservation predictions alone (ProtT5cons 19equal) or BLOSUM62 as additional information (ProtT5beff) were compared to different baselines: BLOSUM62bin relies solely on substitution scores to detect SAVs with deleterious effect, ConSeq 19equal uses only binarized conservation predictions from ConSeq and ConSeq blosum62 combines both, substitution scores and conservation prediction. Further, we evaluate the logistic regression ensemble for Variant Effect Score Prediction without Alignments (VESPA) introduced here and its computationally more efficient counterpart (VESPAl). Additionally, we compare to an existing solution specialized in predicting SAV effect: SNAP2bin. For detailed method descriptions see Methods. ± values mark the 95% confidence interval (Eqn. 12). For each measure, if available, significantly best results are highlighted in bold.

Table S4: Performance comparison for predicting SAV effects on DMS4 (95%)*

Method	$F1_{effect}$ (Eqn. 6)	$F1_{neutral}$ (Eqn. 7)	Q2 (Eqn. 1)	MCC (Eqn. 8)
Random	23.18% ± 1.37	76.72% ± 0.67	64.27% ± 0.76	-0.001 ± 0.017
**Supervised Methods**				
SNAP2bin	42.80% ± 1.0	40.24% ± 1.05	41.55% ± 0.82	0.204 ± 0.012
VESPA	51.57% ± 1.18	70.80% ± 0.78	63.56% ± 0.79	0.346 ± 0.014
VESPAl	55.93% ± 1.23	80.11% ± 0.64	72.59% ± 0.72	**0.405 ± 0.016**
**Rule-Based Methods**				
BLOSUM62bin	41.87% ± 1.06	46.85% ± 1.01	44.47% ± 0.84	0.169 ± 0.014
ProtT5cons 19equal	50.19% ± 1.15	69.54% ± 0.77	62.20% ± 0.82	0.322 ± 0.014
ProtT5beff	53.56% ± 1.26	79.41% ± 0.62	71.47% ± 0.74	0.369 ± 0.016
ConSeq 19equal	46.16% ± 1.11	54.53% ± 0.93	50.70% ± 0.84	0.267 ± 0.012
ConSeq blosum62	50.14% ± 1.18	71.59% ± 0.78	63.81% ± 0.8	0.318 ± 0.015

**DMS4* dataset consisting of binary annotated variants in 4 human proteins (BRAC1, PTEN, TPMT, PPARG). Annotations were created by considering the middle 95% of effect scores as neutral SAVs (Reeb et al. 2020). All variants outside these thresholds were considered effect (neutral SAVs: 11,788, deleterious effect SAVs: 3,516). The results for other thresholds (90%, 99%) can be found in SOM Tables 3,5 (trends are similar). Methods developed here using either binarized conservation predictions alone (ProtT5cons 19equal) or BLOSUM62 as additional information (ProtT5beff) were compared to different baselines: BLOSUM62bin relies solely on substitution scores to detect SAVs with deleterious effect, ConSeq 19equal uses only binarized conservation predictions from ConSeq and ConSeq blosum62 combines both, substitution scores and conservation prediction. Further, we evaluate the logistic regression ensemble for Variant Effect Score Prediction without Alignments (VESPA) introduced here and its computationally more efficient counterpart (VESPAl). Additionally, we compare to an existing solution specialized in predicting SAV effect: SNAP2bin. For detailed method descriptions see Methods. ± values mark the 95% confidence interval (Eqn. 12). For each measure, if available, significantly best results are highlighted in bold.

Table S5: Performance comparison for predicting SAV effects on DMS4 (99%)*

Method	$F1_{effect}$ (Eqn. 6)	$F1_{neutral}$ (Eqn. 7)	Q2 (Eqn. 1)	MCC (Eqn. 8)
Random	10.08% ± 0.76	**89.69% ± 0.19**	**81.51% ± 0.3**	-0.002 ± 0.016
**Supervised Methods**				
SNAP2bin	22.85% ± 0.47	39.21% ± 0.48	32.0% ± 0.37	0.164 ± 0.007
VESPA	31.43% ± 1.25	70.90% ± 0.71	59.14% ± 0.76	0.283 ± 0.012
VESPAl	**37.78% ± 1.48**	81.15% ± 0.54	71.07% ± 0.71	**0.347 ± 0.014**
**Rule-Based Methods**				
BLOSUM62bin	21.90% ± 0.51	44.93% ± 0.47	35.41% ± 0.4	0.118 ± 0.012
ProtT5cons 19equal	31.40% ± 0.64	70.78% ± 0.36	59.01% ± 0.4	0.283 ± 0.011
ProtT5beff	35.96% ± 0.76	80.80% ± 0.28	70.45% ± 0.37	0.316 ± 0.015
ConSeq 19equal	26.98% ± 0.57	57.05% ± 0.44	45.91% ± 0.42	0.235 ± 0.008
ConSeq blosum62	31.20% ± 0.66	73.01% ± 0.33	61.23% ± 0.4	0.268 ± 0.014

**DMS4* dataset consisting of binary annotated variants in 4 human proteins (BRAC1, PTEN, TPMT, PPARG). Annotations were created by considering the middle 99% of effect scores as neutral SAVs (Reeb et al. 2020). All variants outside these thresholds were considered effect (neutral SAVs: 13,506, deleterious effect SAVs: 1,548). The results for other thresholds (90%, 95%) can be found in SOM Tables 3,4 (trends are similar). Methods developed here using either binarized conservation predictions alone (ProtT5cons 19equal) or BLOSUM62 as additional information (ProtT5beff) were compared to different baselines: BLOSUM62bin relies solely on substitution scores to detect SAVs with deleterious effect, ConSeq 19equal uses only binarized conservation predictions from ConSeq and ConSeq blosum62 combines both, substitution scores and conservation prediction. Further, we evaluate the logistic regression ensemble for Variant Effect Score Prediction without Alignments (VESPA) introduced here and its computationally more efficient counterpart (VESPAl). Additionally, we compare to an existing solution specialized in predicting SAV effect: SNAP2bin. For detailed method descriptions see Methods. ± values mark the 95% confidence interval (Eqn. 12). For each measure, if available, significantly best results are highlighted in bold.

Table S6: Pearson correlation between SAV effect prediction and DMS39 experiments*

Method	Mean absolute r_P (Eqn. 10)	Median absolute r_P (Eqn. 10)	Standard Error of mean absolute r_P (Eqn. 10)
**MSA-based**			
DeepSequence	0.49	0.49	0.03
GEMME	0.51	0.53	0.03
pLM-based			
ESM-1v	0.48	0.52	0.03
VESPA	0.49	0.49	0.03
VESPAl	0.46	0.47	0.02

*Data set: DMS39 (39 DMS experiments gathered for development of DeepSequence (Riesselman et al. 2018)) with 135,665 SAV scores. Methods: a) DeepSequence trains an unsupervised model, i.e., no effect score labels are required, using only information from MSAs for each of the DMS experiments individually (Riesselman et al. 2018); b) GEMME uses statistical means together with expert domain knowledge to infer evolutionary trees and conserved sites from MSAs to predict mutational effects (Laine et al. 2019); c) ESM-1v: log-odds ratio of substitution probabilities (see Methods for details) are readily correlated with SAV effect magnitudes without further optimization on any SAV effect data (zero-shot learning) (Meier et al. 2021); d) VESPA (this work): logistic regression ensemble trained on binary effect classification (effect/neutral) using predicted residue conservation (ProtT5cons), substitution scores from BLOSUM62 (Henikoff and Henikoff 1992), and log-odds ratio of substitution probabilities from ProtT5 (Elnaggar et al. 2021); e) VESPAl: "light" version of VESPA using only predicted conservation and BLOSUM62 as input. pLM-based methods (ESM-1v, VESPA, VESPAl) always provide predictions, irrespective of MSA-depth while MSA-based methods (DeepSequence, GEMME) rely on MSA input and provide only predictions above a certain MSA depth/diversity.

Table S7: pLM-based SAV effect prediction: comparison of Spearman correlation for DMS39 scores with and without available MSAs*

Method	Mean absolute r_S (Eqn. 11)	Median absolute r_S (Eqn. 11)	Standard Error of mean absolute r_S (Eqn. 11)
No MSA available			
ESM-1v	0.28	0.25	0.03
VESPA	0.36	0.32	0.04
VESPAl	0.29	0.26	0.04
MSA available			
ESM-1v	0.49	0.53	0.02
VESPA	0.51	0.53	0.02
VESPAl	0.47	0.47	0.02

*Data set: DMS39 (39 DMS experiments gathered for development of DeepSequence (Riesselman et al. 2018)) with 135,665 SAV scores. Methods: a) ESM-1v: substitution probabilities (see Methods for details) are readily correlated with SAV effect magnitudes without further optimization on any SAV effect data (zero-shot learning) (Meier et al. 2021); b) VESPA (this work): logistic regression ensemble trained on binary effect classification (effect/neutral) using predicted residue conservation (ProtT5cons), substitution scores from BLOSUM62 (Henikoff and Henikoff 1992), and log-odds of substitution probabilities from ProtT5 (Elnaggar et al. 2021); c) VESPAl: "light" version of VESPA using only predicted conservation and BLOSUM62 as input. pLM-based methods (ESM-1v, VESPA, VESPAl) always provide predictions, irrespective of MSA-depth while MSA-based methods (DeepSequence, GEMME) rely on MSA input and provide only predictions above a certain MSA depth/diversity. The predictions of pLM-based methods are compared for the substitutions where MSA-based predictions are available (90% of DMS39) to the substitution where there are none (10% of DMS39) (see Table S8).

Table S8: Total number of SAV effect predictions and coverage of DMS scores on DMS39*

Method	Total number of predictions	Coverage of DMS scores
<u>**MSA-based**</u>		
DeepSequence	121,608	89.64%
GEMME	121,608	89.64%
<u>***pLM-based***</u>		
ESM-1v	135,289	99.72%
VESPA	135,289	99.72%
VESPAl	135,289	99.72%

*Data sets: DMS39 (39 DMS experiments gathered for development of DeepSequence (Riesselman et al. 2018)) with 135,665 SAV scores. Methods: a) DeepSequence trains an unsupervised model, i.e., no effect score labels are required, using only information from MSAs for each of the DMS experiments individually (Riesselman et al. 2018); b) GEMME uses statistical means together with expert domain knowledge to infer evolutionary trees and conserved sites from MSAs to predict mutational effects (Laine et al. 2019); c) ESM-1v: log-odds ratio of substitution probabilities (see Methods for details) are readily correlated with SAV effect magnitudes without further optimization on any SAV effect data (zero-shot learning) (Meier et al. 2021); d) VESPA (this work): logistic regression ensemble trained on binary effect classification (effect/neutral) using predicted residue conservation (ProtT5cons), substitution scores from BLOSUM62 (Henikoff and Henikoff 1992), and log-odds ratio of substitution probabilities from ProtT5 (Elnaggar et al. 2021); e) VESPAl: "light" version of VESPA using only predicted conservation and BLOSUM62 as input. pLM-based methods (ESM-1v, VESPA, VESPAl) always provide predictions, irrespective of MSA-depth while MSA-based methods (DeepSequence, GEMME) rely on MSA input and provide only predictions above a certain MSA depth/diversity. The small coverage disparity between pLM-based methods and DMS scores (0.28%) occurred due to minimal deviances of input sequences derived from alignments provided by Riesselman et al. (2018). The column coverage of DMS scores shows the percentage of total DMS annotations for which predictions of each method were available.

References for Supporting Online Material

Ben Chorin A, Masrati G, Kessel A, Narunsky A, Sprinzak J, Lahav S, Ashkenazy H, Ben-Tal N (2020) ConSurf-DB: An accessible repository for the evolutionary conservation patterns of the majority of PDB proteins. Protein Science 29: 258-267. doi: 10.1002/pro.3779

Elnaggar A, Heinzinger M, Dallago C, Rehawi G, Wang Y, Jones L, Gibbs T, Feher T, Angerer C, Steinegger M, Bhowmik D, Rost B (2021) ProtTrans: Towards Cracking the Language of Life's Code Through Self-Supervised Learning. MACHINE INTELLIGENCE 14: 30.

Findlay GM, Daza RM, Martin B, Zhang MD, Leith AP, Gasperini M, Janizek JD, Huang X, Starita LM, Shendure J (2018) Accurate classification of BRCA1 variants with saturation genome editing. Nature 562: 217-222. doi: 10.1038/s41586-018-0461-z

Henikoff S, Henikoff JG (1992) Amino acid substitution matrices from protein blocks. Proceedings of the National Academy of Sciences 89: 10915-10919. doi: 10.1073/pnas.89.22.10915

Kawabata T, Ota M, Nishikawa K (1999) The Protein Mutant Database. Nucleic Acids Research 27: 355-357. doi: 10.1093/nar/27.1.355

Laine E, Karami Y, Carbone A (2019) GEMME: a simple and fast global epistatic model predicting mutational effects. Mol Biol Evol. doi: 10.1093/molbev/msz179

Majithia AR, Tsuda B, Agostini M, Gnanapradeepan K, Rice R, Peloso G, Patel KA, Zhang X, Broekema MF, Patterson N, Duby M, Sharpe T, Kalkhoven E, Rosen ED, Barroso I, Ellard S, Consortium UKMD, Kathiresan S, Myocardial Infarction Genetics C, O'Rahilly S, Consortium UKCL, Chatterjee K, Florez JC, Mikkelsen T, Savage DB, Altshuler D (2016) Prospective functional classification of all possible missense variants in PPARG. Nat Genet 48: 1570-1575. doi: 10.1038/ng.3700

Matreyek KA, Starita LM, Stephany JJ, Martin B, Chiasson MA, Gray VE, Kircher M, Khechaduri A, Dines JN, Hause RJ, Bhatia S, Evans WE, Relling MV, Yang W, Shendure J, Fowler DM (2018) Multiplex assessment of protein variant abundance by massively parallel sequencing. Nat Genet 50: 874-882. doi: 10.1038/s41588-018-0122-z

Meier J, Rao R, Verkuil R, Liu J, Sercu T, Rives A (2021) Language models enable zero-shot prediction of the effects of mutations on protein function. bioRxiv: 2021.07.09.450648. doi: 10.1101/2021.07.09.450648

Reeb J, Wirth T, Rost B (2020) Variant effect predictions capture some aspects of deep mutational scanning experiments. BMC Bioinformatics 21: 107. doi: 10.1186/s12859-020-3439-4

Riesselman AJ, Ingraham JB, Marks DS (2018) Deep generative models of genetic variation capture the effects of mutations. Nat Methods 15: 816-822. doi: 10.1038/s41592-018-0138-4
